# A novel host-targeted inhibitor of clathrin-mediated endocytosis limits spring viremia of carp virus infection

**DOI:** 10.1128/mbio.00453-26

**Published:** 2026-05-29

**Authors:** Lei Liu, Yan Zhou, Huan Wang, Yang Hu, Jiong Chen

**Affiliations:** 1 State Key Laboratory for Quality and Safety of Agro-Products, Ningbo University 47862, Ningbo, China; 2 Laboratory of Biochemistry and Molecular Biology, School of Marine Sciences, Ningbo University 47862, Ningbo, China; 3 Key Laboratory of Applied Marine Biotechnology of Ministry of Education, Ningbo University 47862, Ningbo, China; 4 School of Life Sciences, Huzhou University 117774, Huzhou, China; University of Cape Coast, Cape Coast, Central Region, Ghana

**Keywords:** antiviral, aquatic rhabdovirus, internalization, clathrin, endocytosis, active monomer

## Abstract

Spring viremia of carp virus (SVCV), an aquatic rhabdovirus, causes lethal disease and major economic losses in carp aquaculture, yet no licensed antivirals are available. In this study, we identify the plant-derived diterpenoid quinone cryptotanshinone (CPT) as a potent inhibitor of SVCV that acts by inhibiting clathrin-mediated endocytosis (CME) and attenuating early mitochondrial apoptosis. CPT was well tolerated by epithelioma papulosum cyprinid cells (50% cytotoxic concentration [CC_50_] = 303.2 µM) and suppressed SVCV replication in a dose-dependent manner (50% inhibitory concentration [IC_50_] = 3.2 µM), reducing *nucleoprotein* (SVCV-*N*) gene expression and infectious virion production by >99% at 16 μM. Time-of-addition/removal and virion pre-incubation assays revealed that CPT acts predominantly at early entry or early post-entry steps: CPT did not impair viral attachment but selectively blocked internalization, causing DiO-labeled SVCV particles to remain trapped at the plasma membrane. Mechanistically, CPT inhibited CME, as evidenced by reduced transferrin uptake and redistribution of clathrin heavy chain from the cytosol to the cell surface. CPT also preserved mitochondrial integrity by limiting Bcl-2-associated X protein translocation, cytochrome *c* release, and caspase-9/3 activation. *In vivo*, therapeutic bath treatment with 4 μM CPT significantly lowered viral loads, decreased cumulative mortality, and reduced horizontal transmission in juvenile common carp, whereas prophylactic oral CPT primed type I interferon and interferon-stimulated gene expression, and markedly improved survival after SVCV challenge. Together, these findings define CME-dependent internalization as a druggable entry checkpoint for an aquatic rhabdovirus and highlight CPT as a promising antiviral lead for integrated control of SVCV.

**IMPORTANCE:**

Viral diseases are a major bottleneck to sustainable aquaculture, yet most control strategies still depend on husbandry and culling rather than on mechanism-based antiviral therapy. For spring viremia of carp virus (SVCV), a notifiable rhabdovirus of global concern, it remains unclear whether host entry pathways in fish are realistically “druggable” and how small molecules can be integrated into practical farm interventions. Here, we use cryptotanshinone (CPT) as a chemical probe to demonstrate that clathrin-mediated endocytosis in fish cells can be selectively perturbed to block SVCV internalization, and that this strategy is compatible with immersion and oral delivery in juvenile carp. At the same time, CPT reshapes host responses by stabilizing mitochondrial function and sustaining antiviral signaling, thereby linking a defined entry pathway to cell-death control and innate immunity *in vivo*. These findings open a conceptual route toward host-targeted, entry-focused antivirals for aquatic rhabdoviruses and provide a translational framework for developing environmentally compatible antivirals in aquaculture.

## INTRODUCTION

Aquaculture has emerged as the fastest-growing food-producing sector worldwide and is now indispensable for global food and nutrition security, particularly in low- and middle-income countries where fish represents a major source of high-quality animal protein and essential micronutrients (1). In addition to supplying nutritious food, aquaculture sustains rural livelihoods by creating employment opportunities, stabilizing household income, and alleviating fishing pressure on already overexploited wild stocks (2, 3). However, the long-term sustainability of this sector is increasingly threatened by infectious diseases, especially viral pathogens, which can cause sudden mass mortalities and devastating economic losses in the absence of effective therapeutics or widely deployed vaccines (4). Among the notifiable aquatic animal diseases recognized by the World Organisation for Animal Health, spring viremia of carp (SVC) is caused by the spring viremia of carp virus (SVCV), an enveloped, negative-sense, single-stranded RNA rhabdovirus that primarily infects cyprinid fishes, including common carp, koi, grass carp, and goldfish, as well as several other susceptible species (5). First reported in European river systems, SVCV has since been detected in Asia, the Middle East, and the Americas, where it can induce acute systemic disease in juvenile fish with case fatality rates frequently approaching 70%–90%, thereby posing a major constraint to carp aquaculture (5, 6). Despite progress in experimental vaccine development, no globally licensed and broadly used vaccine or antiviral drug is currently available, and control still relies largely on conventional biosecurity measures such as stock movement restrictions, surveillance, quarantine, and stamping-out policies (7). Consequently, the continued risk of SVCV outbreaks underscores an urgent need to develop novel, affordable antiviral strategies that can be integrated into existing health management programs to enhance the sustainability and resilience of carp aquaculture.

The life cycle of enveloped viruses can be broadly divided into three interconnected stages: (i) viral entry, during which virions bind to specific receptors or attachment factors on the host cell surface, undergo conformational rearrangements, and deliver their genomes across cellular membranes; (ii) replication and gene expression, whereby the viral genome hijacks host biosynthetic machinery to drive transcription, translation, and genome amplification; and (iii) assembly and release, during which progeny virions are assembled and exit infected cells via budding or lytic pathways to initiate subsequent rounds of infection (8, 9). Among these steps, entry represents a critical bottleneck that shapes host and tissue tropism and largely determines the efficiency of productive infection, because successful attachment and internalization are prerequisites for downstream replication. Enveloped viruses commonly exploit host endocytic pathways, including clathrin-mediated endocytosis (CME), macropinocytosis, and caveolae- or lipid raft–mediated endocytosis, to gain access to intracellular compartments—a strategy that not only facilitates efficient entry but also promotes immune evasion by shielding viral components from early recognition (10). As a result, the entry stage has emerged as an attractive target for antiviral intervention: blockade of attachment, endocytosis, or membrane fusion can prevent infection at an early step, limit viral amplification, and reduce the likelihood of excessive inflammatory responses (11–13), while the dependence of diverse viruses on conserved host entry machineries provides opportunities for broad-spectrum inhibition (14–16). Consistent with this concept, previous studies have shown that several aquatic rhabdoviruses, including SVCV, *Micropterus salmoides* rhabdovirus (MSRV), and infectious hematopoietic necrosis virus (IHNV), enter host cells predominantly via low-pH-dependent clathrin-mediated endocytosis (17–19), and a small-molecule rhodanine derivative designed to interfere with this pathway is reported to exert potent anti-IHNV activity by targeting the early stages of infection (20).

Plant-derived bioactive compounds remain a rich source of antiviral leads, as their structurally diverse secondary metabolites can target multiple steps of the viral life cycle, although poor solubility and limited bioavailability still hamper their clinical translation (8). Accumulating evidence indicates that numerous phytochemicals, including isoliquiritigenin, dihydroartemisinin, bis-benzylisoquinoline alkaloids such as (S,S)-(+)-tetrandrine, and the neolignan honokiol, exert potent antiviral and immunomodulatory activities against both mammalian and aquatic viruses (21–23). Despite these advances, the precise molecular mechanisms by which most plant-derived compounds restrict infection, particularly in the context of fish rhabdoviruses, remain incompletely defined, and it is often unclear whether they act primarily on viral entry, intracellular replication, or host antiviral signaling pathways.

Cryptotanshinone (CPT), a lipophilic tanshinone-type diterpenoid quinone from *Salvia miltiorrhiza* (Danshen), has shown antiviral and immunomodulatory activities in diverse mammalian-virus systems (24–26). Because many enveloped fish viruses, including SVCV, rely on endocytic uptake routes such as CME for productive infection, we asked whether CPT could serve as a host-oriented inhibitor of SVCV. Here, we show that CPT restricts SVCV infection in epithelioma papulosum cyprinid (EPC) cells and juvenile common carp by blocking CME-dependent internalization at early infection and by mitigating infection-associated mitochondrial stress and cell death while supporting innate antiviral responses. Together, these data nominate CPT as a lead compound for developing green antivirals and highlight viral entry checkpoints as tractable intervention nodes in aquaculture.

## RESULTS

### CPT is not cytotoxic at antiviral concentrations and blocks SVCV infection *in vitro*

**Fig 1:**
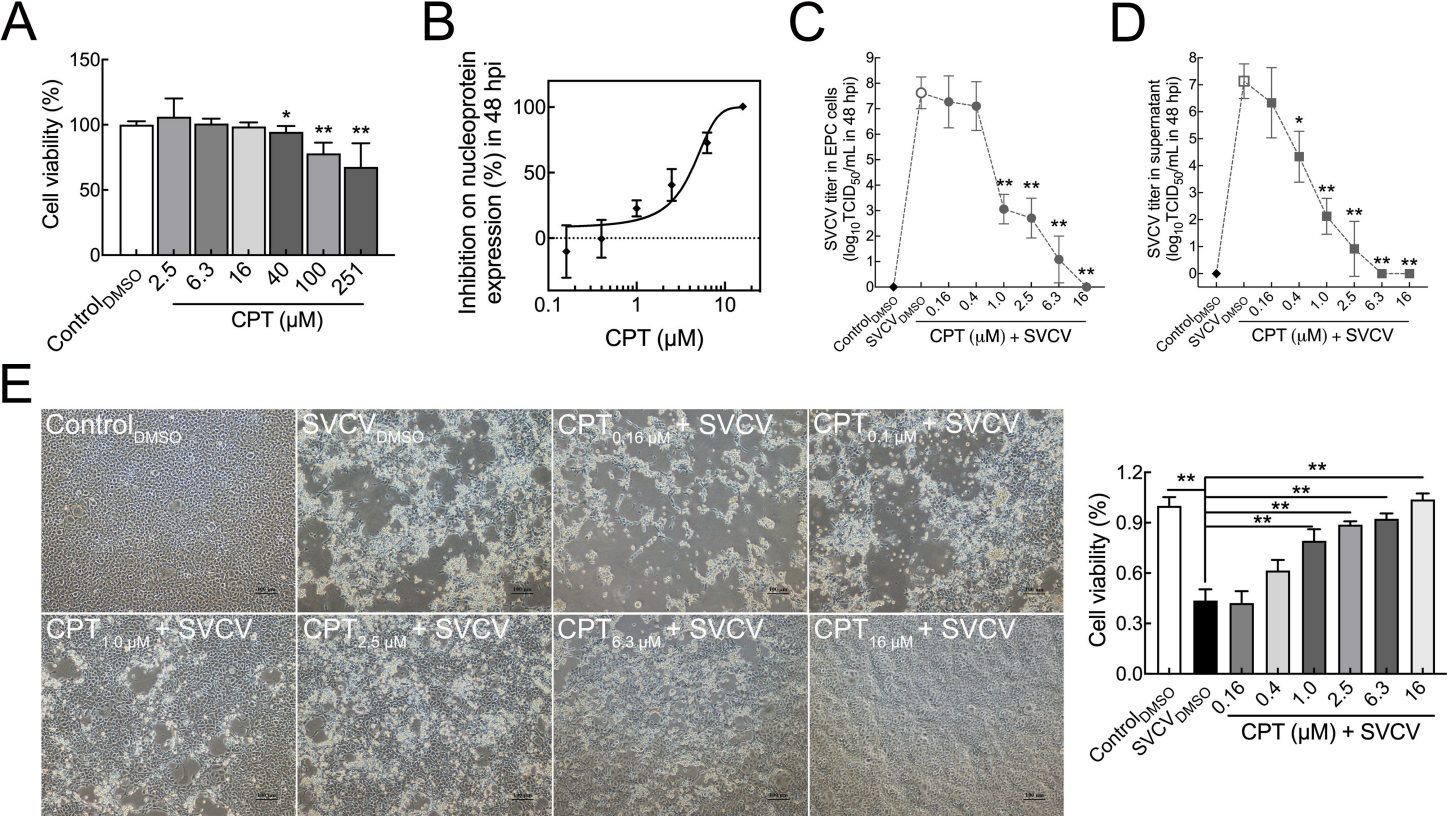
CPT is minimally cytotoxic and potently inhibits SVCV replication in EPC cells. (**A**) Cytotoxicity of CPT in EPC cells. Cells were treated with the indicated concentrations of CPT for 48 h, and cell viability was measured by CCK-8 assay. (**B**) Dose-dependent inhibition of SVCV-*N* expression by CPT. EPC cells were infected with SVCV and treated with CPT at the indicated concentrations. Viral N mRNA levels at 48 hpi were quantified by RT-qPCR and expressed as percent inhibition relative to the virus + DMSO group. (**C and D**) Effect of CPT on the production of infectious SVCV. At 48 hpi, cell-associated virus and virus released into the culture supernatant were collected and titrated by TCID_50_ assay. Treatment with 16 μM CPT almost abolished cell-associated virus and completely suppressed detectable virus in the supernatant. (**E**) CPT alleviates SVCV-induced cytopathic effects and restores cell viability. Representative phase-contrast images of EPC cells in the indicated treatment groups at 48 hpi (left), and quantification of cell viability by CCK-8 assay (right). Data are presented as mean ± SD. Statistical significance was determined by one-way ANOVA with appropriate *post hoc* tests; *P < 0.05, **P < 0.01.

To define a non-cytotoxic working range, EPC cells were exposed to CPT for 48 h, and viability was quantified by CCK-8 assay. CPT showed low cytotoxicity (50% cytotoxic concentration = 303.2 µM) and did not measurably affect viability at ≤16.0 µM (Fig. 1A). Within this range, CPT inhibited SVCV infection in a dose-dependent manner, reducing viral *nucleoprotein* (SVCV-*N*) gene expression at 48 h post-infection (hpi) with a 50% inhibitory concentration (IC_50_) of 3.2 μM and >99.5% maximal inhibition at 16 μM (Fig. 1B). Consistently, 50% tissue culture infective dose (TCID_50_) assays revealed a marked loss of infectious progeny: 16 μM CPT nearly eliminated cell-associated titers and fully prevented infectious virus release into supernatants (Fig. 1C and D). CPT also alleviated SVCV-induced cytopathic effects and restored cell viability at ≥2.5 µM (Fig. 1E). Collectively, CPT is well tolerated at antiviral doses and potently blocks SVCV replication and progeny production *in vitro*.

### CPT prevents SVCV-induced mitochondrial dysfunction

**Fig 2:**
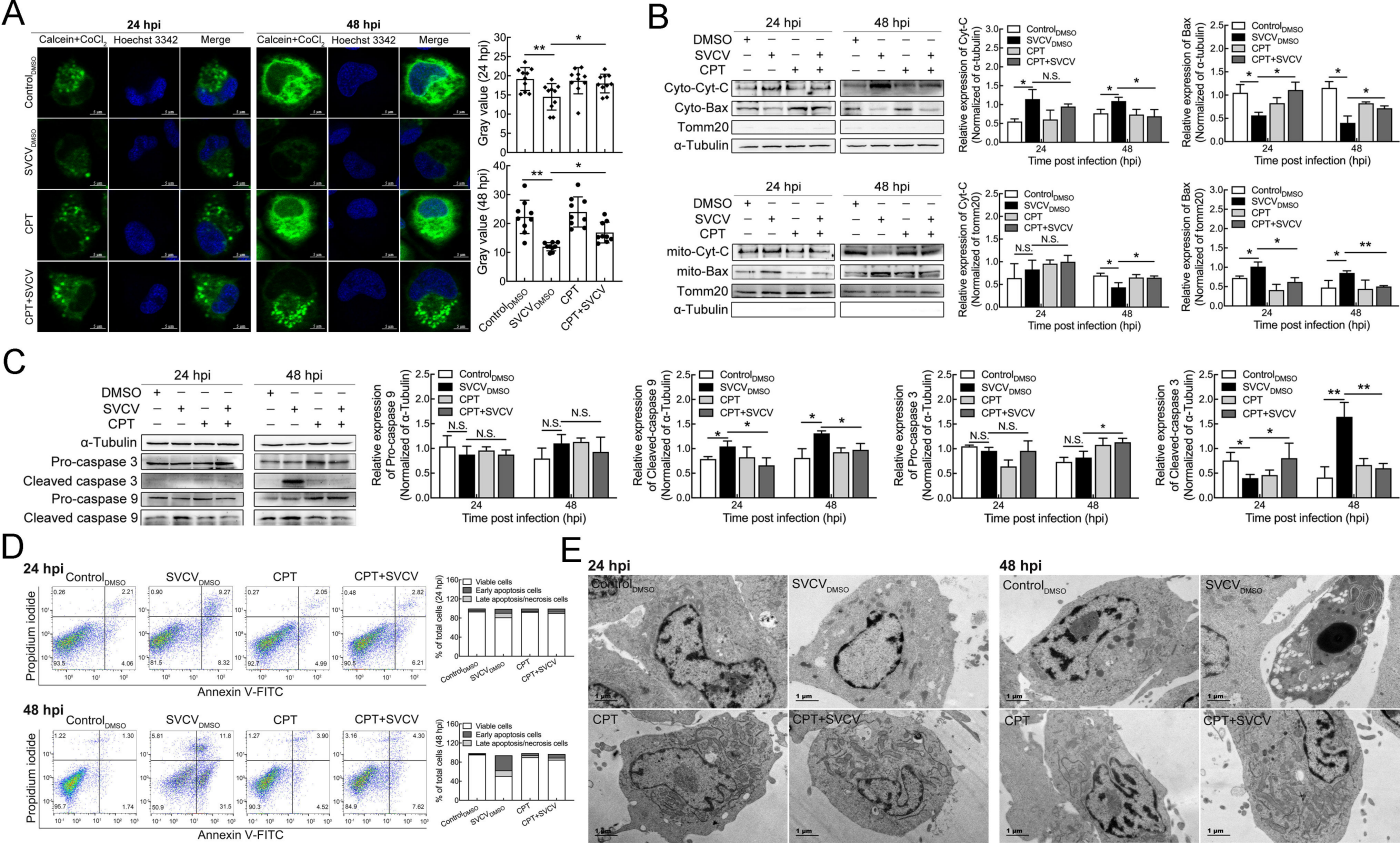
CPT preserves mitochondrial integrity and inhibits Bcl-2-associated X protein (Bax)-dependent intrinsic apoptosis in SVCV-infected EPC cells. (**A**) Calcein/CoCl_2_ quenching assay of EPC cells at 24 and 48 hpi showing that CPT restores mitochondrial calcein fluorescence reduced by SVCV infection. Quantification of gray values is shown on the right. (**B**) Immunoblot analysis of cytochrome *c* (Cyt-C) and Bax in cytosolic and mitochondrial fractions. SVCV promotes Cyt-C release and Bax accumulation in mitochondria, whereas CPT reverses these changes. Densitometric analysis is shown on the right. (**C**) Immunoblot analysis of pro- and cleaved caspase-3 and caspase-9. SVCV activates the mitochondrial caspase cascade, which is suppressed by CPT. Quantification of cleaved caspases is shown on the right. (**D**) Annexin V/PI flow-cytometric analysis of apoptosis. CPT markedly reduces SVCV-induced apoptosis and increases the proportion of viable cells at 24 and 48 hpi. (**E**) Transmission electron microscopy of EPC cells. SVCV causes mitochondrial fragmentation, cristae disruption, and nuclear abnormalities, whereas CPT-treated infected cells largely retain normal ultrastructure. Scale bars, 1 μm. Data are presented as mean ± SD. Statistical significance was determined by one-way ANOVA with appropriate *post hoc* tests; *P < 0.05, **P < 0.01.

To assess whether CPT protects EPC cells from SVCV-triggered intrinsic apoptosis, we examined apoptosis-associated morphology and mitochondrial function. SVCV infection (48 h) induced chromatin condensation/nuclear fragmentation and microtubule collapse, whereas these changes were largely prevented by CPT (Fig. S1A). We then quantified ΔΨm and permeability. JC-1 staining revealed progressive mitochondrial depolarization in infected cells at 24–48 h, while CPT preserved ΔΨm (Fig. S1B). In addition, calcein/CoCl₂ quenching assays showed that SVCV promoted mitochondrial permeability transition (loss of mitochondrial calcein signal), which was significantly reversed by CPT (Fig. S2A and B). Consistently, the assays detected bright mitochondrial fluorescence in healthy cells and strong quenching after SVCV infection, reflecting the opening of the permeability transition pore, while CPT significantly restored calcein signal in infected cells (Fig. 2A). Flow cytometry corroborated these results, showing reduced calcein fluorescence after infection and a CPT-dependent restoration at both 24 and 48 h (Fig. S3). Together, CPT prevents SVCV-induced mitochondrial depolarization and permeability transition.

### CPT inhibits Bcl-2-associated X protein-dependent intrinsic apoptosis and rescues SVCV-infected cells

We next tested whether CPT interferes with Bax-mediated mitochondrial apoptotic signaling. Subcellular fractionation/immunoblotting showed that SVCV infection drove Bax translocation to mitochondria and promoted Cyt-C release into the cytosol at 24–48 h, whereas CPT largely retained Bax in the cytosol and restored mitochondrial Cyt-C (Fig. 2B). Consistently, Bax-GFP displayed increased mitochondrial colocalization upon SVCV infection, which was markedly reduced by CPT (Fig. S4). Downstream, SVCV activated the mitochondrial caspase cascade (cleaved caspase-9 and caspase-3), and CPT substantially blunted activation of both caspases (Fig. 2C). Annexin V/PI analysis further showed that CPT dose-dependently rescued cell viability during infection, maintaining >90% viable cells at 16 μM across 24–48 h (Fig. 2D). Ultrastructurally, transmission electron microscopy confirmed that CPT mitigated infection-associated mitochondrial damage (fragmentation and cristae loss) and cytoplasmic vacuolization, preserving overall organellar integrity (Fig. 2E).

### CPT interferes with the early stages of SVCV infection by blocking viral internalization

**Fig 3:**
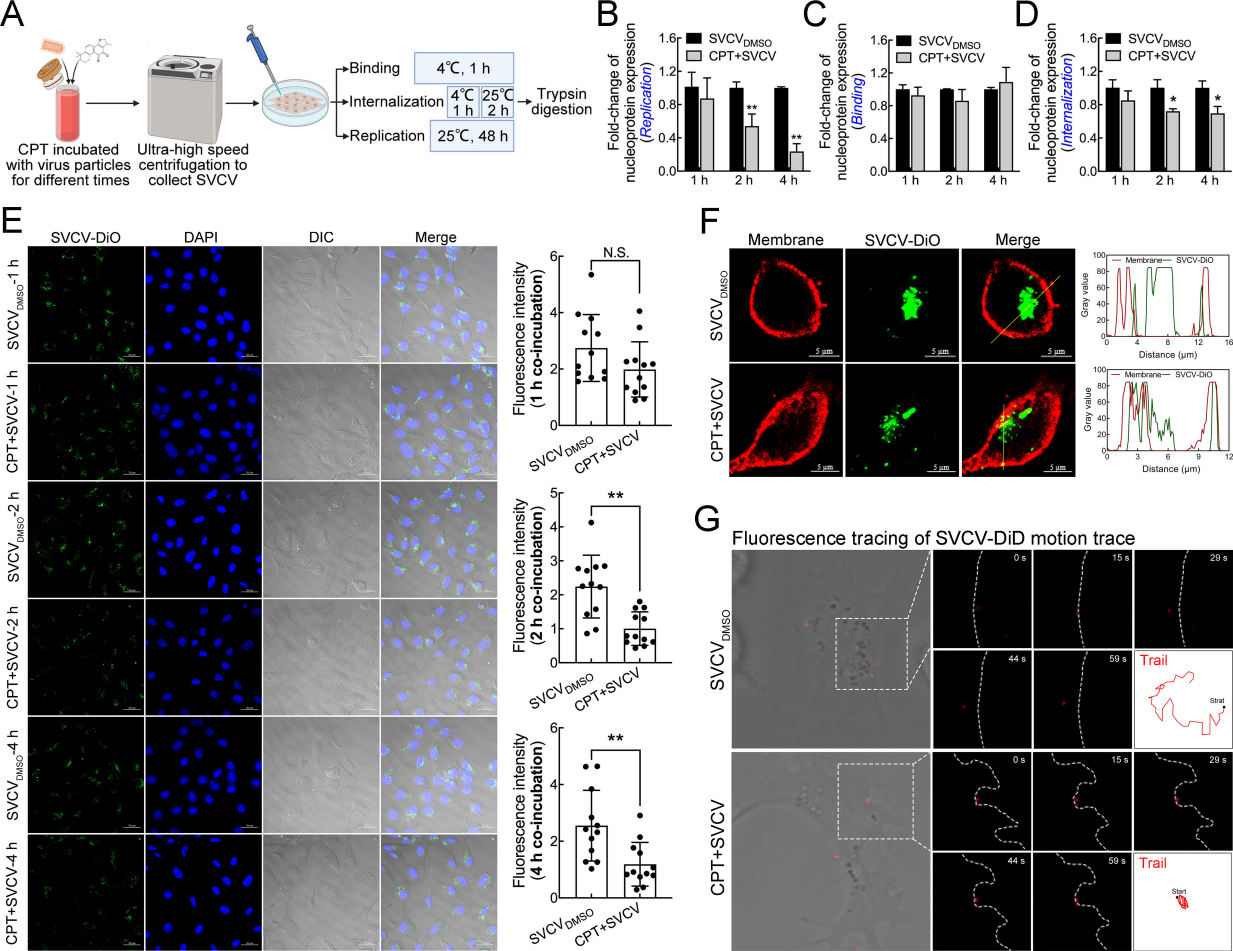
Virion pre-incubation with CPT impairs subsequent SVCV internalization. (**A**) Schematic diagram of the experimental design. SVCV particles were incubated with CPT for the indicated times, purified by ultracentrifugation, and then used to infect EPC cells for binding, internalization, or replication assays. (**B–D**) RT-qPCR analysis of SVCV-*N* gene expression after infection with DMSO-treated (SVCV_DMSO_) or CPT-treated (CPT + SVCV) virions. Effects of CPT pre-incubation on viral replication (**B**), attachment at 4°C (**C**), and internalization after shift to 25°C (**D**) are shown. (**E**) Confocal images of EPC cells infected with DiO-labeled SVCV (green) after pre-incubation of virions with DMSO or CPT for 1, 2, or 4 h. Nuclei were stained with DAPI (blue), and differential interference contrast (DIC) images are shown. Quantification of intracellular DiO fluorescence intensity is shown on the right. (**F**) Colocalization of DiO-labeled SVCV (green) with DiD-labeled plasma membrane (red) in cells infected with DMSO- or CPT-treated virions. Line-scan profiles of membrane and viral fluorescence along the indicated lines are shown to the right. (**G**) Representative motion tracks of DiD-labeled SVCV particles in live EPC cells after virion pre-incubation with DMSO or CPT. Data are presented as mean ± SD. Statistical significance was determined by one-way ANOVA with appropriate *post hoc* tests; *P < 0.05, **P < 0.01.

To map the stage targeted by CPT, we performed time-of-addition (ToA) and time-of-removal (ToR) assays. The two curves intersected at 5 hpi (range, 4–6 hpi), indicating that CPT acts mainly during early entry/early post-entry rather than late assembly or release (Fig. S5A and B). We then asked whether virion pre-incubation with CPT altered subsequent infectivity. SVCV particles were pre-incubated with CPT (2–4 h), washed to remove unbound compound, and subjected to adsorption, internalization, and replication assays (Fig. 3A). CPT pre-treatment of virions reduced subsequent infection (decreased SVCV-*N* gene expression and CPE), yet did not affect viral adsorption to EPC cells (Fig. 3B and C). In contrast, internalization was markedly impaired, as shown by reduced levels of internalized viral RNA and by fluorescent tracking of DiO-labeled virions, which accumulated at the cell surface and colocalized with the plasma membrane marker DiD (Fig. 3D through G; Movies S1 and S2). Thus, the virion pre-incubation data are consistent with an effect of CPT on virions that compromises efficient internalization without disrupting initial attachment.

**Fig 4:**
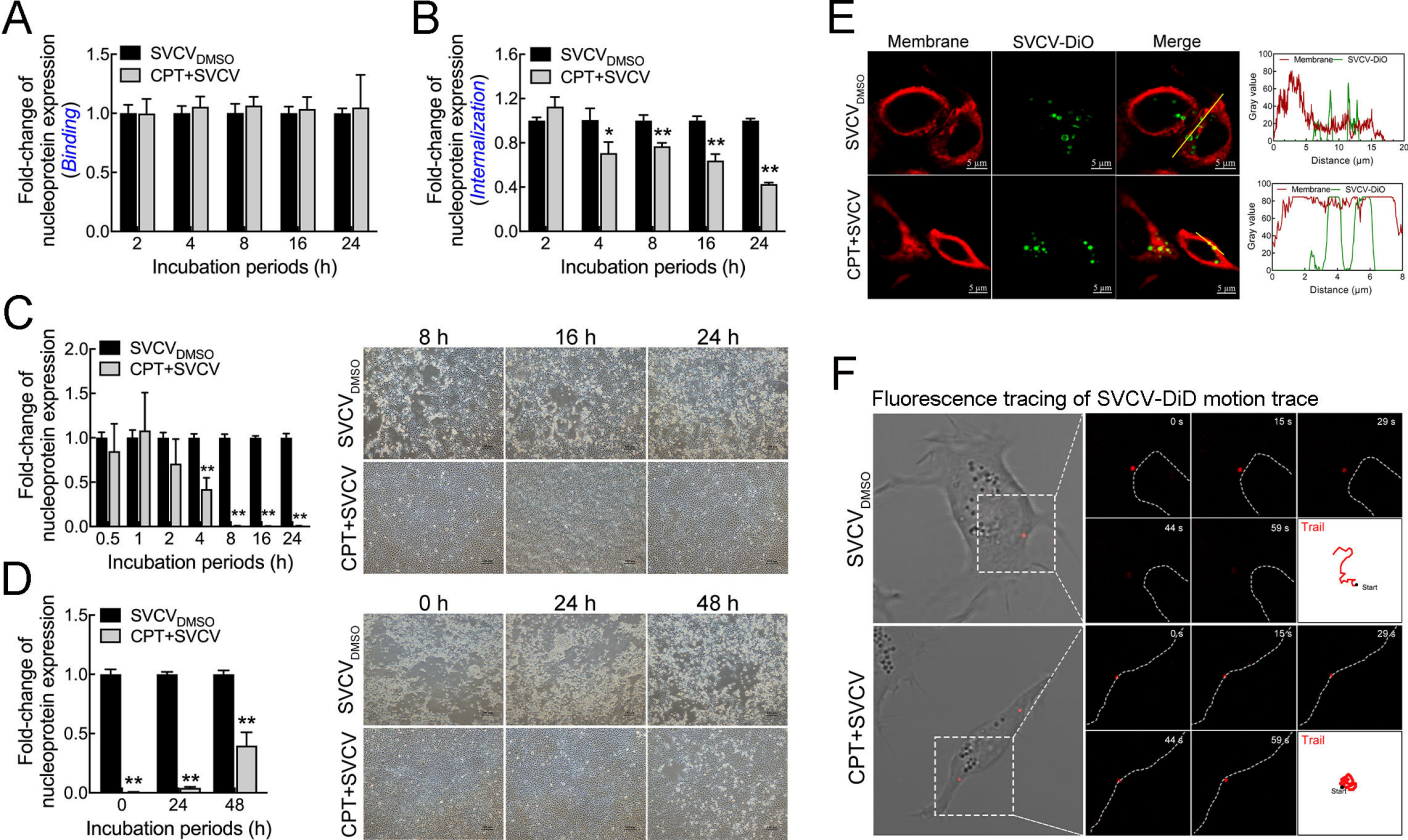
CPT pretreatment of EPC cells blocks SVCV internalization and replication in a reversible manner. (**A**) Effect of CPT pretreatment on SVCV binding. *Nucleoprotein* mRNA level was measured by RT-qPCR and expressed as fold change relative to SVCV_DMSO_. (**B**) Effect of CPT pretreatment on SVCV internalization. After CPT pretreatment for the indicated times, cells were infected with SVCV, shifted to 25°C for 2 h, treated with trypsin, and SVCV-*N* gene expression was quantified. (**C**) Effect of CPT pretreatment on SVCV replication. EPC cells were pre-incubated with CPT for the indicated times, infected with SVCV, and SVCV-*N* gene expression was determined at 48 hpi (left). Representative bright-field images at 8, 16, and 24 hpi (right) show that CPT pretreatment ≥4 h greatly reduces CPE. (**D**) Reversibility of CPT-mediated inhibition. After 24 h CPT pretreatment, cells were washed and cultured in drug-free medium for the indicated periods before SVCV infection. SVCV-*N* gene expression (left) and CPE (right) were evaluated at 48 hpi. (**E**) Colocalization of DiO-labeled SVCV (green) with DiD-labeled plasma membrane (red) in DMSO- or CPT-pretreated cells. Line-scan plots to the right show overlapping fluorescence peaks in CPT-pretreated cells, indicating virions remaining at the membrane. (**F**) Representative motion traces of DiD-labeled SVCV particles on live EPC cells pretreated with DMSO or CPT. In control cells, virions move inward from the cell surface, whereas in CPT-pretreated cells, they remain largely immobilized at the plasma membrane. Data are presented as mean ± SD. Statistical significance was determined by one-way ANOVA with appropriate *post hoc* tests; *P < 0.05, **P < 0.01.

We next examined whether CPT also targets the host entry machinery. EPC cells were pretreated with CPT, washed, and then challenged with SVCV. CPT pretreatment did not alter viral adsorption at any time point (Fig. 4A), but reduced internalization in a pretreatment-time-dependent manner (Fig. 4B) and strongly suppressed downstream replication and CPE after ≥4 h pretreatment (Fig. 4C). This effect was reversible: after drug washout, the inhibition of viral gene expression progressively declined and CPE re-emerged (Fig. 4D), consistent with a non-permanent modulation of host entry competence. Imaging of DiO-labeled SVCV on DiD-stained cells also revealed an internalization defect: in DMSO-treated cells, virions rapidly penetrated into the cytoplasm with little colocalization with the plasma membrane, whereas in cells pretreated with CPT for 24 h, many virions remained associated with the cell surface and failed to enter the cell (Fig. 4E). Line-scan analysis confirmed extensive overlap between DiO and DiD signals in CPT-pretreated cells, and live-cell imaging revealed that, unlike in control cells where attached virions quickly moved inward, a fraction of virions in CPT-treated cells stayed immobilized on the membrane throughout the observation period (Fig. 4F; Movies S3 and S4). Therefore, our findings indicate that both virion pre-incubation with CPT and host-cell pretreatment impair SVCV internalization, thereby limiting early infection and subsequent viral replication.

### CPT inhibits clathrin-mediated endocytosis required for SVCV entry

**Fig 5:**
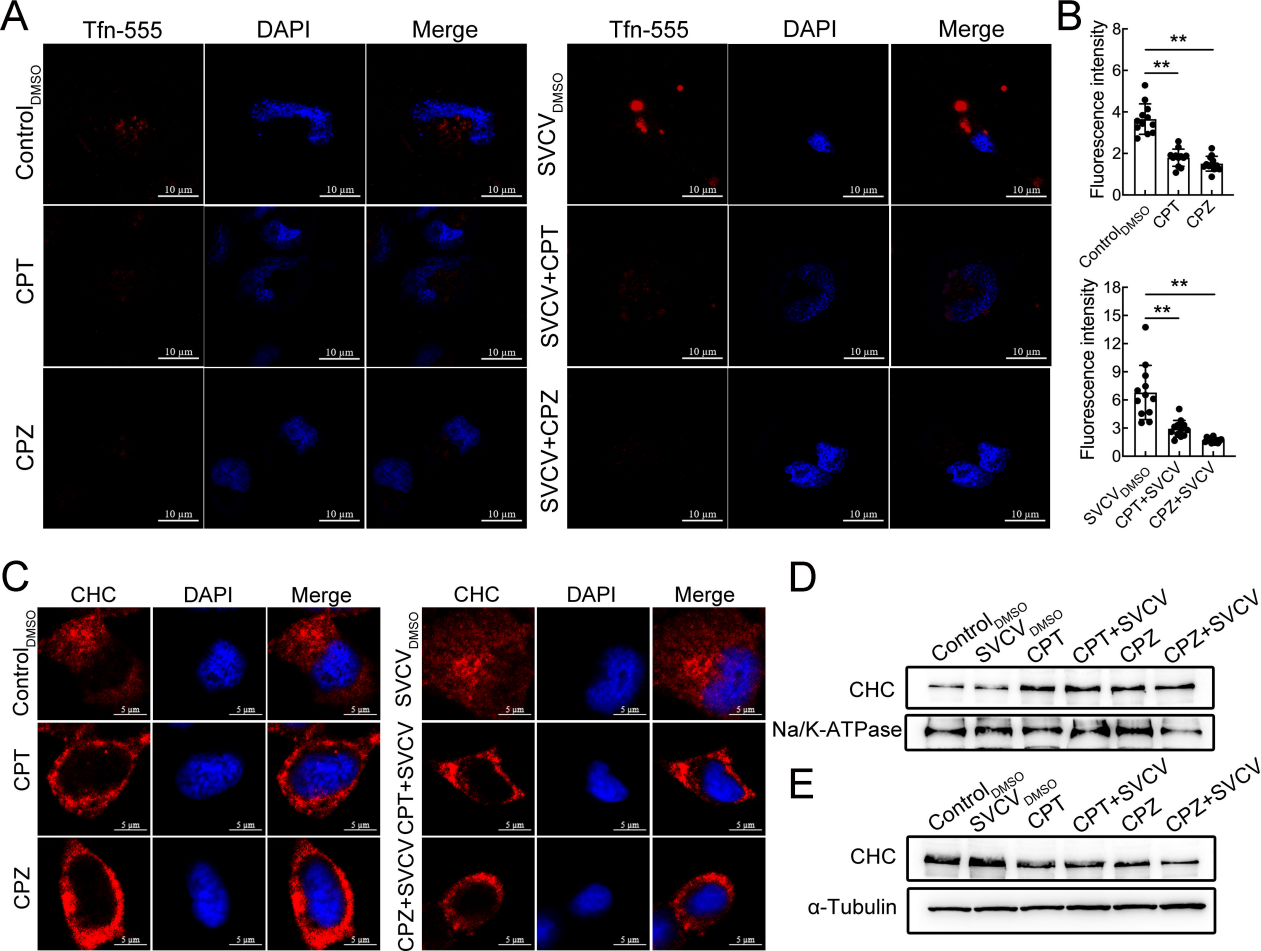
CPT inhibits clathrin-mediated endocytosis in EPC cells. (**A**) Confocal images of Tfn-555 uptake in EPC cells treated with DMSO, CPT, or CPZ in the absence or presence of SVCV. Nuclei were stained with DAPI. (**B**) Quantification of intracellular Tfn-555 fluorescence intensity in the indicated groups. Both CPT and CPZ significantly reduce Tfn-555 uptake, with or without SVCV infection. (**C**) Immunofluorescence staining of CHC showing its distribution in Control_DMSO_, SVCV_DMSO_, CPT, CPT + SVCV, CPZ, and CPZ + SVCV cells. CPT and CPZ promote peripheral clustering of CHC at the plasma membrane. (**D and E**) Immunoblot analysis of CHC in plasma membrane (D; Na/K-ATPase as marker) and cytosolic (E; α-tubulin as marker) fractions. CPT and CPZ increase membrane-associated CHC while decreasing cytosolic CHC. Data are presented as mean ± SD. **P < 0.01.

CME is a major route for SVCV entry, and transferrin (Tfn) uptake via the Tfn receptor serves as a classical marker of CME activity (27, 28). To determine whether CPT interferes with this pathway, EPC cells were pretreated with CPT for 24 h, infected with SVCV, and then incubated with Tfn-555; chlorpromazine (CPZ), a specific CME inhibitor, was included as a positive control. In SVCV-exposed cells, Tfn-555 uptake was increased compared with the uninfected control group, indicating that SVCV stimulates host CME (Fig. 5A). In contrast, both CPT and CPZ markedly reduced intracellular Tfn-555 fluorescence in the presence or absence of infection. Quantitative analysis showed that the mean fluorescence intensity decreased from 3.66 ± 0.70 in control cells to 1.80 ± 0.40 and 1.51 ± 0.34 in CPT- and CPZ-treated cells, respectively. Under SVCV-infected conditions, CPT and CPZ pretreatment reduced Tfn-555 uptake by 56.89% and 74.76%, respectively, relative to the SVCV_DMSO_ group (Fig. 5B), consistent with a strong inhibitory effect of CPT on CME in EPC cells. Consistent with established signatures of CME blockade, clathrin heavy chain (CHC) redistributed from a diffuse cytosolic pattern to accumulation at the plasma membrane. In our experiments, CHC immunofluorescence was relatively uniform in Control_DMSO_ and SVCV_DMSO_ cells, whereas CPT or CPZ treatment caused CHC to redistribute and cluster along the cell periphery (Fig. 5C). Moreover, biochemical fractionation revealed that CHC was predominantly detected in the cytosolic fraction with only low levels at the plasma membrane in Control_DMSO_ and SVCV_DMSO_ cells, while CPT- or CPZ-treated cells displayed increased CHC abundance in membrane fractions accompanied by a corresponding decrease in the cytosolic pool (Fig. 5D and E). Together, these results indicate that CPT disrupts CME in EPC cells, a process that is required for efficient SVCV entry.

### CPT bath treatment protects juvenile carp from lethal SVCV challenge and limits horizontal transmission

**Fig 6:**
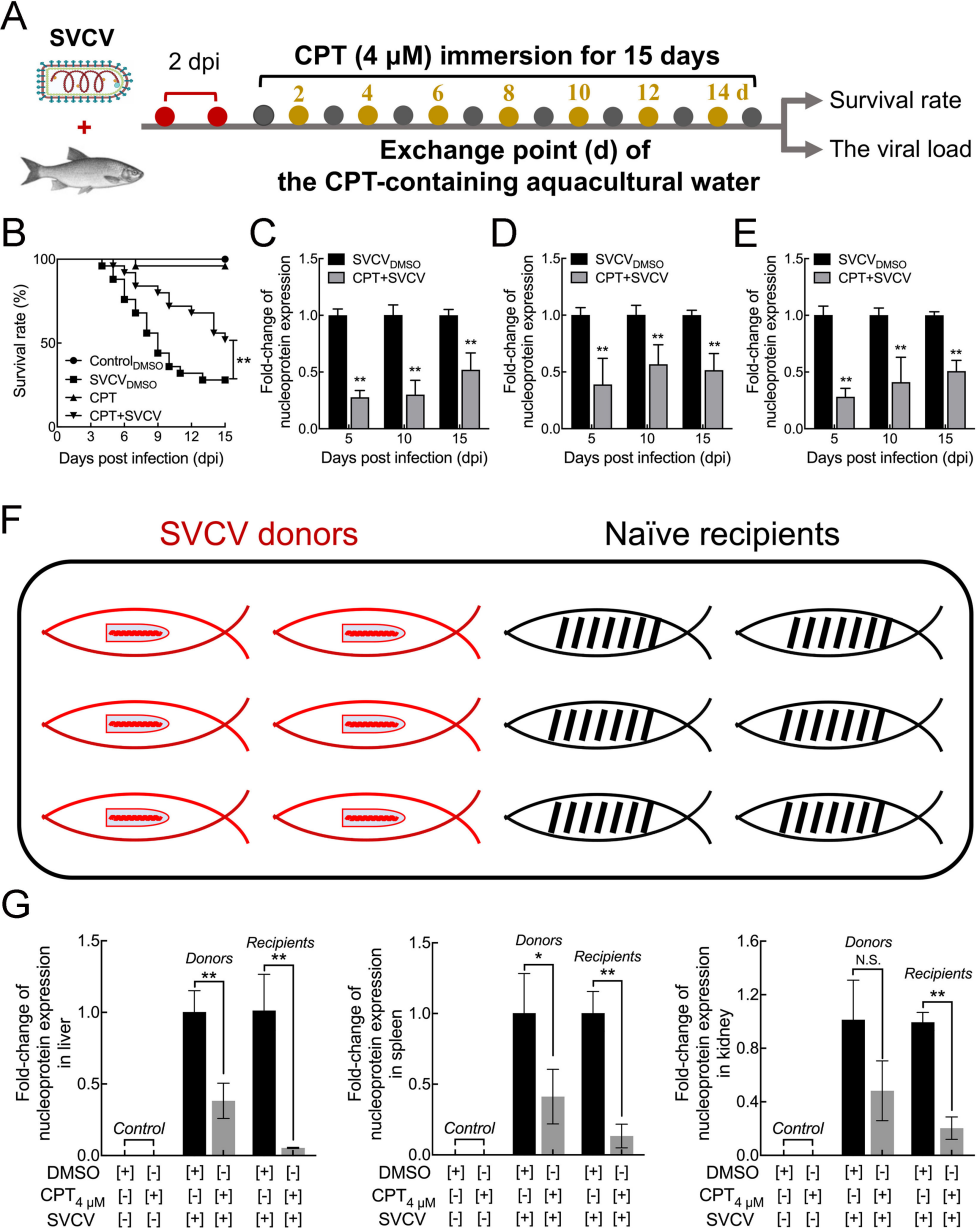
CPT bath treatment protects juvenile carp from lethal SVCV challenge and limits horizontal transmission. (**A**) Schematic of the *in vivo* immersion experiment. Juvenile carp were intraperitoneally injected with SVCV (1 × 10^8^ TCID_50_/mL), and 2 dpi were transferred to static tanks containing 4 μM CPT or 0.0025% DMSO. Fish were maintained for 15 days, with CPT/DMSO-containing water renewed every 2 days, and sampled at 5, 10, and 15 dpi. (**B**) Kaplan–Meier survival curves of Control_DMSO_, SVCV_DMSO_, CPT, and CPT + SVCV groups (*n* = 50 per group). (**C–E**) Fold change of SVCV-*N* gene expression in liver (**C**), spleen (**D**), and kidney (**E**) of SVCV_DMSO_ and CPT + SVCV fish at the indicated dpi, as determined by RT-qPCR (*n* = 6 fish per group per time point). (**F**) Schematic of the cohabitation assay used to evaluate horizontal transmission. SVCV-infected donor fish, treated with DMSO or CPT (4 μM), were cohabited with naïve recipient fish in aerated containers with daily transfer to fresh treatment water. (**G**) SVCV-*N* gene expression in liver, spleen, and kidney of donor and recipient fish after 144 h of cohabitation, expressed as fold change relative to the uninfected control group. Data in bar graphs are mean ± SD. *P < 0.05, **P < 0.01 (two-tailed Student’s *t* test).

Based on preliminary toxicity tests, we next evaluated whether CPT could protect juvenile common carp from high-dose SVCV challenge. Fish were injected intraperitoneally with SVCV and, at 2 dpi, were transferred to water containing 4 μM CPT (Fig. 6A). Although a small number of fish died in the CPT-only group, cumulative mortality did not differ from the DMSO control, indicating that 4 μM CPT is well tolerated under our experimental conditions (Fig. 6B). In the high-dose SVCV group, mortality began at 4 days post-exposure and reached 56% by day 9, with a final cumulative mortality of 72%. By contrast, therapeutic bath treatment with CPT reduced cumulative mortality to 48%, corresponding to a 24% increase in survival compared with SVCV-infected fish without CPT treatment (Fig. 6C). To link this survival benefit to viral control, we quantified SVCV loads by RT-qPCR. CPT-treated infected fish exhibited significantly lower viral burdens in liver, spleen, and kidney at 5, 10, and 15 days post-exposure than untreated infected controls (Fig. 6D through F). Notably, in cohabitation experiments, recipient fish exposed to water from CPT-treated, SVCV-infected donors carried markedly lower SVCV titers than those cohabiting with DMSO-treated, SVCV-infected donors (Fig. 6G), indicating that CPT bath treatment not only protects juvenile carp against lethal SVCV challenge but also substantially reduces horizontal transmission within the population.

### Prophylactic oral CPT administration enhances resistance to SVCV by priming antiviral gene expression

**Fig 7:**
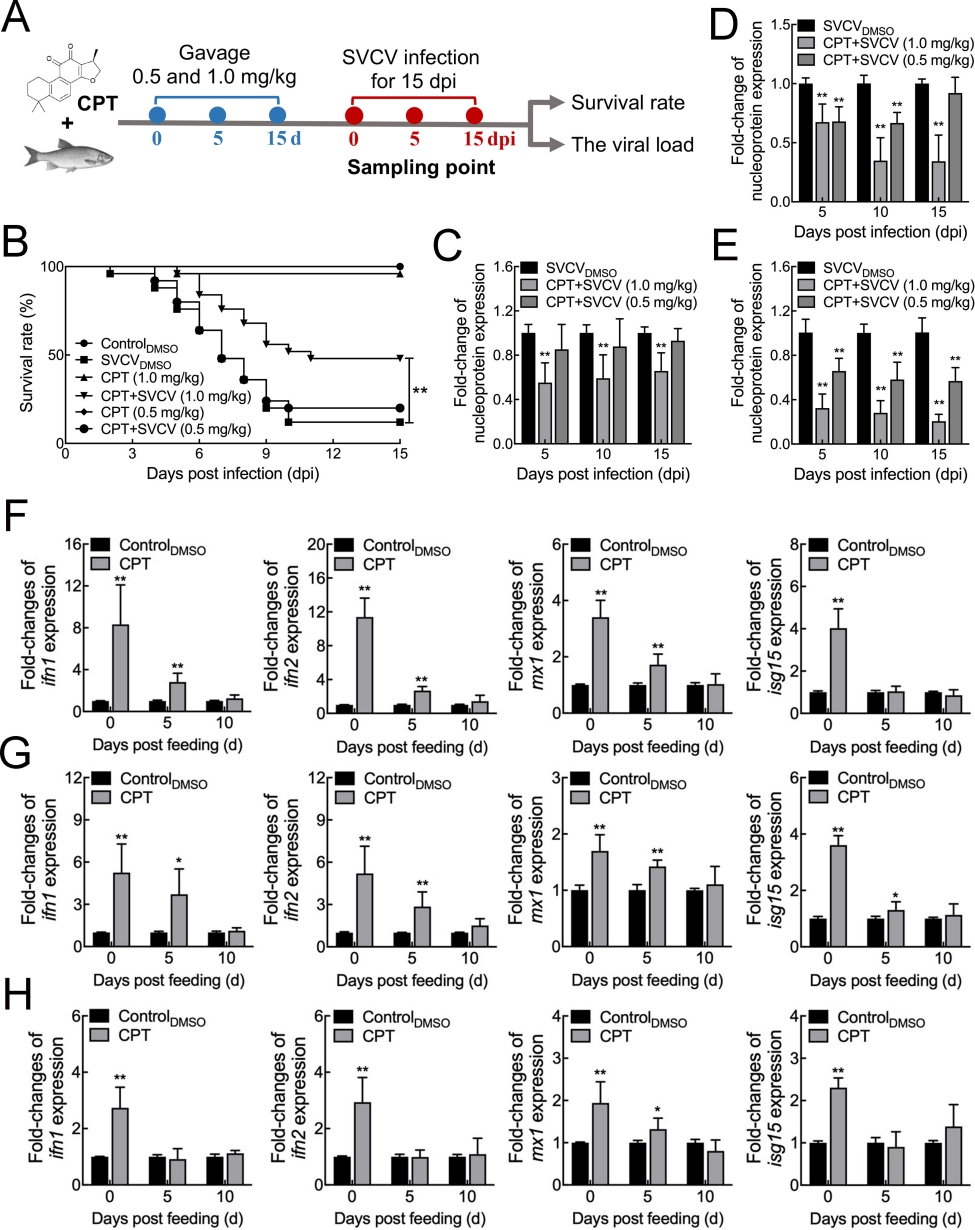
Prophylactic oral CPT administration enhances resistance to SVCV by priming antiviral gene expression. (**A**) Experimental scheme. Juvenile carp were orally gavaged with CPT at 0.5 or 1.0 mg/kg body weight on days 0, 5, and 10, and then challenged with SVCV (1 × 10^8^ TCID_50_/mL) and monitored for 15 dpi. (**B**) Kaplan–Meier survival curves of Control_DMSO_, SVCV_DMSO_, CPT (0.5 or 1.0 mg/kg), and CPT + SVCV groups (*n* = 50 fish per group). (**C–E**) Fold change of SVCV-*N* gene expression in liver (**C**), spleen (**D**), and kidney (**E**) of SVCV_DMSO_ and CPT + SVCV fish at 5, 10, and 15 dpi, determined by RT-qPCR (*n* = 6 per group per time point). (**F–H**) Expression of antiviral genes in CPT-gavaged but uninfected fish. Juveniles were gavaged with CPT at 1.0 mg/kg, and liver (**F**), spleen (**G**), and kidney (**H**) were sampled at 0, 5, and 10 days after the last gavage. Relative mRNA levels of *ifn1*, *ifn2*, *mx1,* and *isg15* were measured by RT-qPCR and are shown as fold change versus Control_DMSO_ fish (*n* = 6 per group per time point). Data are presented as mean ± SD. *P < 0.05, **P < 0.01 (two-tailed Student’s *t* test versus corresponding control).

Because viral diseases in aquaculture often have long incubation periods and are difficult to control once clinical outbreaks occur, we next examined whether CPT could be used prophylactically to enhance resistance to SVCV. Juvenile carp were orally gavaged with CPT at 0.5 or 1.0 mg/kg body weight once every 5 days over a 15-day period (days 0, 5, and 10), followed by SVCV challenge (Fig. 7A). In the untreated SVCV-infected group, mortality started at 2 dpi, whereas deaths in the 0.5 and 1.0 mg/kg CPT groups were delayed until 4 and 5 dpi, respectively. At the end of the experiment, survival rates were 12% in the SVCV group, 20% in the 0.5 mg/kg group, and 48% in the 1.0 mg/kg group, indicating that high-dose CPT increased survival by 36 percentage points relative to untreated infected fish (Fig. 7B). Consistent with these survival data, viral load measurements demonstrated that both CPT doses reduced SVCV replication in liver, spleen, and kidney to varying degrees, with the 1.0 mg/kg group exhibiting significant suppression of SVCV-*N* gene expression at all examined time points (Fig. 7C through E). To investigate the mechanism underlying this prophylactic protection, we profiled antiviral gene expression in CPT-treated fish. Immediately after the 15-day gavage regimen (day 0), liver tissues from the 1.0 mg/kg group displayed robust induction of type I interferons and interferon-stimulated genes: *ifn1* and *ifn2* were upregulated by 8.3-fold and 11.4-fold, respectively, and myxovirus resistance 1 (*mx1*) and interferon-stimulated gene 15 (*isg15*) were increased more than threefold relative to control (Fig. 7F). In spleen and kidney, *ifn1* and *ifn2* were also significantly elevated, albeit to a lesser extent. The CPT-induced transcriptional activation gradually waned over time but remained detectable: 5 days after cessation of gavage, antiviral genes in the spleen were still clearly upregulated compared with controls, whereas in the liver only *ifn1*, *ifn2,* and *mx1* remained significantly higher, and in the kidney significant induction persisted for *mx1* (1.32-fold) (Fig. 7G and H). Additionally, the temporal pattern of interferon and ISG induction is consistent with a priming effect, in which oral CPT administration establishes a heightened antiviral state that enhances host resistance to subsequent SVCV challenge.

## DISCUSSION

CPT, a tanshinone-type diterpenoid from Salvia miltiorrhiza, is recognized as a pleiotropic natural product with anti-inflammatory, antitumor, and antiviral activities, yet its potential application in aquaculture remains largely unexplored. In fish farming, viral epizootics caused by rhabdoviruses, iridoviruses, and betanodaviruses continue to compromise production despite improvements in husbandry and vaccination (29). Environmentally compatible antivirals and immunomodulators, particularly plant-derived small molecules, are increasingly viewed as attractive tools for disease control in aquatic animals (29, 30). Several natural products, including artemisinin and its derivatives, tetrandrine, arctigenin, and coumarin analogs, are reported to exert strong inhibitory effects against SVCV and other fish rhabdoviruses in cell culture and fish models (22, 31–33). Our study adds CPT to this panel and identifies it as a highly active anti-SVCV compound that is effective both *in vitro* and *in vivo*, significantly lowering viral burden, limiting tissue injury, and improving survival in juvenile carp while exhibiting minimal toxicity at therapeutically relevant concentrations.

Our findings reinforce viral entry as a strategic stage for intervention. Inhibitors that disrupt receptor-triggered uptake can abort infection before genome delivery and may reduce peak viral loads, tissue damage, and transmission when used therapeutically or prophylactically (34, 35). CME and related endocytic routes are widely exploited by fish and mammalian viruses, and pharmacological CME inhibitors can markedly reduce infection by rhabdoviruses and other enveloped viruses (17, 19, 36–41). Within this framework, our time-of-addition and virion-preincubation experiments place CPT among entry- or early post-entry-acting inhibitors: CPT is most effective when present during the initial uptake window, blocks internalization without affecting attachment, and phenocopies chlorpromazine by reducing transferrin uptake and redistributing clathrin heavy chain, consistent with CME inhibition.

In addition to its effects on entry, CPT exerts host-directed regulation of mitochondrial function and apoptosis that likely contributes to its antiviral efficacy. In porcine alveolar macrophages, CPT suppresses PRRSV replication by downregulating CD163, inhibiting STAT3 activation, and limiting IL-10 overproduction, thereby restoring antiviral competence (26, 42). More recently, CPT is reported to restrict bovine viral diarrhea virus (BVDV) propagation through a combination of direct virion inactivation and interference with replication, again pointing to dual virucidal and cell-targeted activities (43). Our results in EPC cells provide an additional mechanistic dimension: CPT maintains mitochondrial membrane potential and permeability, prevents Bax translocation to mitochondria, reduces Cyt-C release, and attenuates caspase-9/3 activation—events that closely parallel those described for other host-oriented antivirals, such as the curcumin analog EF-24 against *Siniperca chuatsi* rhabdovirus (44). By constraining excessive intrinsic apoptosis, CPT is likely to preserve the mitochondrial antiviral signaling (MAVS) platform and sustain RIG-I-like receptor (RLR)–interferon regulatory factor 3/7 (IRF3/7) signaling, thereby supporting type I interferon production in response to SVCV. The immunomodulatory effects observed *in vivo* fit well with current models of fish innate antiviral immunity and with the activities of other plant-derived immunostimulants. Type I interferons and their downstream ISGs are central components of antiviral defense in teleosts and are induced by a broad spectrum of RNA viruses (45). Here, prophylactic oral administration of CPT primes juvenile carp, leading to sustained upregulation of *ifn1*, *ifn2*, *mx1,* and *isg15* in liver, spleen, and kidney and conferring significant protection against subsequent SVCV challenge. Taken together, these findings are consistent with CPT being part of a growing class of host-directed, immunomodulatory natural-product antivirals that combine direct suppression of viral replication with durable modulation of the host innate immune landscape.

Despite the efficacy observed here, several practical limitations should be addressed before CPT can be translated into an aquaculture antiviral. CPT is highly lipophilic and poorly water-soluble, which can lead to variable exposure and complicate large-scale immersion or feed delivery; accordingly, DMSO-free, farm-compatible formulations (e.g., lipid/nanocrystal-based approaches) will likely be required to achieve predictable dosing (46, 47). In addition, CPT/tanshinones can modulate drug-metabolizing enzymes, raising the possibility of off-target effects or interactions with other therapeutants commonly used on farms (48). Finally, advancement toward field use will require fish pharmacokinetic and residue-depletion studies to support dose optimization and withdrawal periods consistent with international guidance for aquatic species.

**Fig 8:**
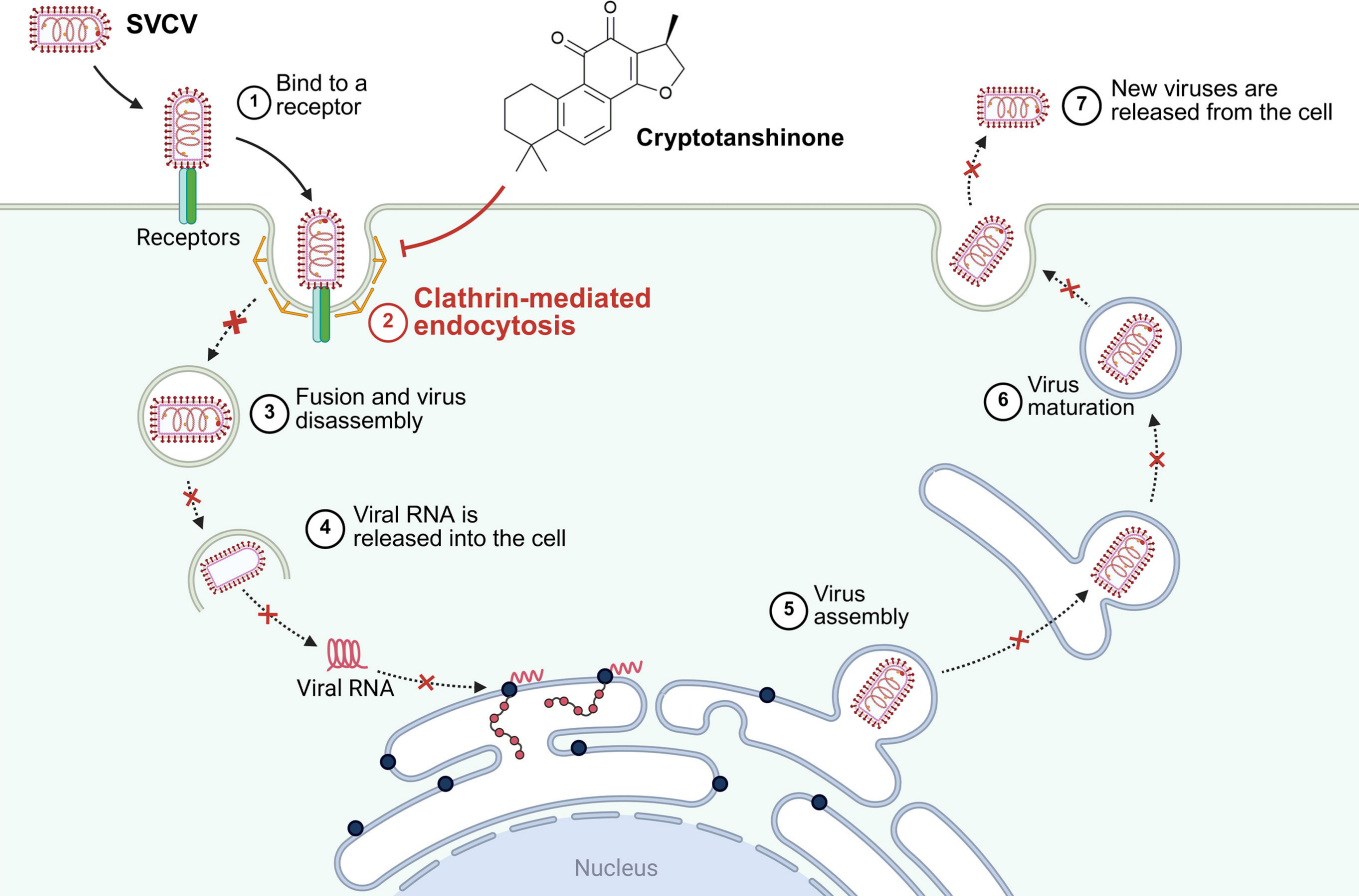
Schematic model of CPT inhibition of SVCV infection. SVCV binds to receptors at the surface of host cells and normally enters via CME, followed by endosomal fusion and disassembly, release of viral RNA into the cytoplasm, replication and transcription, assembly of progeny virions in association with intracellular membranes, maturation in the secretory pathway, and release of new virions. CPT targets the CME step at the host–virus interface, preventing efficient internalization of attached virions and thereby blocking downstream events of the replication cycle. By acting at this early entry checkpoint, CPT limits SVCV replication and spread *in vitro* and *in vivo*. Created in BioRender (L. Lei, 2026, https://BioRender.com/qz5h92g).

In conclusion, this study identifies CPT as a potent host-oriented inhibitor of SVCV infection. Our data indicate that CPT acts predominantly at an early stage of infection by disrupting clathrin-mediated endocytosis at the host–virus interface and thereby limiting viral internalization. In parallel, CPT-treated infected cells display preserved mitochondrial integrity and reduced Bax translocation/caspase activation, consistent with attenuation of intrinsic apoptosis and improved cellular fitness; however, whether this mitochondrial protection reflects a direct action of CPT or occurs secondarily to reduced early infection will require further investigation. *In vivo*, both therapeutic immersion and prophylactic oral administration of CPT reduce viral loads, limit tissue damage, improve survival, and diminish horizontal transmission in juvenile carp, and oral dosing is accompanied by a primed type I interferon/ISG program. Together, these findings nominate CPT as a promising lead compound for the development of green antivirals in aquaculture and highlight viral entry pathways and mitochondrial stress/death checkpoints as complementary host processes that may be leveraged to control rhabdovirus diseases in farmed fish (Fig. 8).

## MATERIALS AND METHODS

### Experimental fish, cell culture, and virus infection

Juvenile common carp (*Cyprinus carpio*) with an average body weight of 2.94 ± 0.26 g and total length of 4.93 ± 0.48 cm were obtained from Fish and Rice Agricultural Development Co., Ltd. (Guizhou Province, China). Fish were initially maintained in a recirculating freshwater system at 20°C under standard laboratory conditions. Before antiviral challenge experiments, virus-free carp were transferred to a static-water system and acclimated for 2 weeks at 15°C to minimize handling stress and to ensure a uniform baseline health status.

EPC cells, derived from the skin of fathead minnow (*Pimephales promelas*), were a gift from Prof. Yong Zhou (the Yangtze River Fisheries Research Institute, Chinese Academy of Fishery Sciences), and cultured in Medium 199 (M199; HyClone) supplemented with 10% heat-inactivated fetal bovine serum (FBS; Every Green), 100 U/mL penicillin, and 0.1 mg/mL streptomycin (Beyotime). Cells were maintained at 25°C in a humidified atmosphere containing 5% CO_2_.

The laboratory strain SVCV-0504 (originally isolated from common carp) was propagated in EPC cells. Virus stocks were prepared by infecting EPC monolayers and harvesting the culture supernatant when extensive CPE was observed. SVCV was purified from clarified supernatants by sucrose gradient ultracentrifugation (Beckman 70Ti rotor) at 100,000 × *g* for 3 h, as previously described (49). The purified virus pellet was resuspended in sterile phosphate-buffered saline (PBS) and stored at −80°C until use. Viral titers were determined on EPC cells in 96-well plates by the TCID_50_ assay and calculated according to the Reed–Müench method; the stock used in this study had a titer of 1.63 × 10^8^ TCID_50_/mL. For fluorescence tracking experiments, purified SVCV particles resuspended in PBS were labeled with DiO (#V22886, Invitrogen) or DiD (#V22887, Invitrogen) at a final concentration of 10 μM in the dark at room temperature for 10 min, as described previously (17). Excess dye was removed using a NAP-5 filtration column (GE Healthcare, USA). The labeled virus preparations (DiO-SVCV and DiD-SVCV) were aliquoted and stored at −80°C until further use.

### Reagents

CPT (CAS 35825-57-1, purity ≥ 98%; #C408350, Aladdin) was dissolved in 100% dimethyl sulfoxide (DMSO; Beyotime) to prepare a 50 mg/mL stock solution. Aliquots were stored at −20°C, protected from light, and used within 3 months of preparation. CPZ (#HY-12708, MedChemExpress) was dissolved in DMSO to a final concentration of 10 mM. The stock solution was stored at −20°C in the dark and used within 1 month.

### Cell viability assay (cytotoxicity)

For cytotoxicity assessment, EPC cells were seeded into 96-well plates and incubated at 25°C until they formed a stable monolayer. Cells were then exposed to serial dilutions of CPT (up to 251 μM) or to 0.01% (vol/vol) DMSO as a vehicle control for 48 h in triplicate wells. A previous study showed that DMSO at concentrations up to 0.1% (vol/vol) was neither cytotoxic to EPC cells nor inhibitory to SVCV replication (50); accordingly, the final DMSO concentration was maintained at 0.01% in the 16 μM CPT group and in the corresponding control. Cell viability was determined using the Cell Counting Kit-8 (CCK-8; Beyotime) according to the manufacturer’s instructions. Briefly, 10 μL of CCK-8 solution was added to each well, and plates were incubated for 2 h at 25°C. Absorbance at 450 nm was measured using an ELX800 microplate reader (BioTek Instruments, Canada). The absorbance values, proportional to the number of viable cells, were used to calculate cell viability relative to the DMSO control.

### *In vitro* inhibition of SVCV by CPT

EPC cells were seeded into 96-well or 12-well plates and allowed to form a confluent monolayer for 16 h at 25°C. Cells were then infected with SVCV at 1.0 × 10^4^ TCID_50_/mL in the presence of CPT (0–16 μM) or 0.01% (vol/vol) DMSO. After 48-h incubation at 25°C, antiviral efficacy was evaluated by quantifying SVCV-*N* gene expression and infectious virus production. For analysis of viral replication, total RNA was extracted from EPC cells and subjected to RT-qPCR to determine SVCV-*N* gene expression. In parallel, cell-associated virus and culture supernatants were collected and titrated on EPC cells in 96-well plates to determine infectious titers using the Reed–Müench method. In addition, cell viability under each treatment condition was assessed by CCK-8 assay as described above, providing an integrated measure of antiviral activity and cytoprotection.

### Apoptosis detection

For nuclear morphology and cytoskeletal analysis, SVCV-infected and/or CPT-treated EPC cells grown on glass coverslips were washed three times with PBS and incubated at room temperature in the dark for 30 min with 4′,6-diamidino-2-phenylindole (DAPI; 1 mg/L, Beyotime) and DiI (5 mg/mL, Beyotime). After staining, coverslips were mounted with antifade medium and examined using an upright fluorescence microscope (NI-U, Nikon) to visualize chromatin condensation, nuclear fragmentation, and cell morphology. Apoptosis and related mitochondrial events were further quantified by flow cytometry. Briefly, EPC cells were harvested, washed with cold PBS, and stained with an annexin V/propidium iodide (PI) apoptosis detection kit (Vazyme) according to the manufacturer’s instructions to distinguish viable, early apoptotic, and late apoptotic/necrotic cells. For analysis of ΔΨm, cells were stained with the JC-1 fluorescent probe (Solarbio), and for mitochondrial permeability transition, cells were loaded with calcein acetoxymethyl ester (calcein-AM; Beyotime) followed by CoCl_2_ quenching, in each case according to the manufacturers’ protocols. After staining, nuclei were counterstained with DAPI, where indicated. Fluorescence signals were acquired on a MACSQuant Analyzer 10 flow cytometer (Miltenyi Biotec), and at least 10,000 events were collected per sample for quantitative analysis.

### Time-of-addition and time-of-removal assays

ToA and ToR assays were performed to determine the stage of the SVCV life cycle targeted by CPT. Briefly, EPC cells were seeded into 12-well plates and cultured to a confluent monolayer. Cells were then infected with SVCV at 1.0 × 10^4^ TCID_50_/mL and allowed to adsorb for 1 h at 25°C. After adsorption, the inoculum was removed, the cells were washed with M199 medium, and fresh medium was added. For the ToA assay, CPT was added at a final concentration of 16 μM at different times post-infection and maintained in the culture medium until 48 hpi, when samples were collected. For the ToR assay, cells were treated with 16 μM CPT immediately after adsorption (0 h), and the CPT-containing medium was replaced with drug-free M199 at the indicated time points. In all experiments, cells treated with 0.01% DMSO served as the virus-infected control group. At 48 hpi, total RNA was extracted, and SVCV-*N* gene expression was quantified by RT-qPCR to assess the impact of CPT addition or removal on viral replication.

### Viral infectivity and cellular antiviral resistance

Purified SVCV particles were incubated with 16 μM CPT at 25°C for up to 4 h. After incubation, excess CPT was removed by ultracentrifugation, and the virus pellet was resuspended in fresh medium. EPC cells were then infected with the CPT-treated or DMSO-treated virus at 1 × 10^4^ TCID_50_/mL and incubated at 25°C for 48 h.

For binding assays, CPT- or DMSO-treated SVCV was added to EPC monolayers at 1 × 10^8^ TCID_50_/mL and incubated at 4°C for 1 h to allow attachment without internalization. Cells were then washed twice with ice-cold PBS to remove unbound virions. For internalization assays, after the 1-h binding step at 4°C, cells were shifted to 25°C and incubated for an additional 2 h to permit internalization. Non-internalized virions were removed by brief treatment with trypsin, followed by washes with ice-cold PBS. After the indicated incubations, CPE was recorded using an inverted microscope, and total RNA was extracted for RT-qPCR analysis of SVCV-*N* gene expression.

To assess the impact of CPT on cellular antiviral resistance, EPC cells were directly treated with 16 μM CPT for up to 24 h. Following pretreatment, cells were washed with PBS and re-incubated in virus-containing medium to evaluate SVCV replication, binding, and internalization as described above. In parallel, an additional set of CPT-pretreated cells was washed and then cultured in drug-free medium for a further 24 h prior to infection, to examine the reversibility of CPT-induced antiviral resistance, as previously described (22).

### Virus tracking and analysis

To examine the effect of CPT on SVCV internalization, EPC cells with or without CPT pretreatment were incubated with DiO-labeled SVCV (DiO-SVCV) at 25°C. After incubation, uninternalized virions were removed by brief trypsin treatment, and cells were fixed with 4% paraformaldehyde for 20 min at room temperature. In a parallel set of samples, EPC cells exposed to DiO-SVCV were fixed without trypsinization to visualize both surface-bound and internalized virus. Following fixation, cells were washed with PBS and sequentially stained with DiD to label the plasma membrane and DAPI (100 ng/mL; Beyotime) to label nuclei for 5 min each in the dark, with three PBS washes between steps (5 min per wash). Viral entry and colocalization of DiO-SVCV with the DiD-labeled plasma membrane were observed using a confocal laser scanning microscope (LSM 880, Carl Zeiss, Germany), and fluorescence intensity profiles and colocalization were quantified using ImageJ.

To monitor single-particle entry of SVCV in live cells, EPC cells were cultured on glass-bottom confocal dishes and incubated with DiD-labeled SVCV (DiD-SVCV). Dishes were placed in a temperature-controlled chamber (25°C) on the LSM 880 confocal microscope. DiD fluorescence was excited at 633 nm, and time-lapse images were acquired at 3-s intervals for up to 10 min. Trajectories and velocities of individual viral particles were analyzed using the particle-tracking plugins in ImageJ. The dynamic behavior of fluorescently labeled virions was further characterized by plotting mean-squared displacement (MSD) as a function of time.

### *In vivo* experiments

Acute toxicity assessment of CPT. Before performing antiviral trials, the toxicity of CPT was evaluated by both immersion and oral gavage. Groups of 10 juvenile carp were maintained in 80 L tanks containing UV-sterilized, well-aerated freshwater at 15°C under static conditions. Fish were exposed to CPT at the indicated concentrations and monitored for mortality and behavioral abnormalities over the observation period, following established guidelines for acute toxicity testing in fish. These data were used to determine non-lethal doses for subsequent antiviral experiments.

Therapeutic immersion against SVCV infection. For *in vivo* antiviral efficacy assays, juvenile carp were intraperitoneally injected with 100 μL of SVCV (1.0 × 10^8^ TCID_50_/mL). Two days post-infection (dpi), fish were randomly allocated into two groups (50 fish per group) and subjected to bath treatment. One group was immersed in 0.0025% (vol/vol) DMSO as a vehicle control, and the other group was immersed in 4 μM CPT. Fish were held in 80 L static tanks with continuous aeration at 15°C and fed commercial dry pellets at 0.1% of body weight three times daily. Survival and clinical signs were monitored daily for 15 days. Every 48 h, fish were transferred to fresh water containing renewed CPT (4 μM) or DMSO (0.0025%), and subsets of fish were sampled to determine viral loads in the liver, spleen, and kidney by RT-qPCR.

Prophylactic oral administration of CPT. Groups of 50 juvenile carp received CPT by oral gavage at 0.5 mg/kg or 1.0 mg/kg body weight once every 5 days over a 15-day period (days 0, 5, and 10). To assess the impact of CPT on innate immune responses independently of infection, additional groups of six fish each (without SVCV challenge) were gavaged with CPT at 0.5 mg/kg or 1.0 mg/kg and then sampled for gene expression analysis. At the end of the 15-day gavage regimen, all remaining fish were intraperitoneally injected with 100 μL of SVCV (1.0 × 10^8^ TCID_50_/mL) and maintained in 80 L tanks containing UV-sterilized, well-aerated freshwater at 15°C under static conditions for 15 dpi. Survival and clinical signs were recorded daily, and viral loads in target tissues were measured at designated time points over the 15-day period.

For tissue collection, fish were anesthetized with 3-aminobenzoic acid ethyl ester methanesulfonate (MS-222) at a final concentration of 20.0 g/m³. Liver, spleen, and kidney were aseptically excised, immediately frozen in liquid nitrogen, and stored at −80°C until RNA extraction. The expression of four innate antiviral genes was analyzed by RT-qPCR as described below. All *in vivo* experiments were performed in duplicate (two independent tanks per treatment).

### Cohabitation analysis on viral horizontal transmission

Cohabitation experiments to assess horizontal transmission of SVCV were performed as previously described with minor modifications (20). Briefly, donor juveniles were either intraperitoneally injected with SVCV (5.0 × 10^9^ TCID_50_/mL) or treated with 0.0025% (vol/vol) DMSO. After a 2-day infection period, six donor fish were transferred into individual aerated challenge containers containing 4 μM CPT. Following a 30 min pre-treatment, six naïve recipient juveniles were added to each container to initiate cohabitation. Donor and recipient fish were transferred every 24 h to new containers with freshly prepared treatment solutions. After 144 h of cohabitation, surviving recipient fish were euthanized and sampled, and SVCV loads were determined by RT-qPCR. Each treatment was performed in triplicate (three independent containers per treatment), and the entire cohabitation experiment was repeated twice.

### RNA extraction, reverse transcription, and RT-qPCR

Total RNA was extracted from EPC cells and fish tissues using TRIzol reagent (TaKaRa) according to the manufacturer’s instructions. Residual genomic DNA was removed by digestion with RNase-free DNase I (Promega). First-strand cDNA was synthesized from 1 μg of total RNA using the HiScript II 1st Strand cDNA Synthesis Kit (+gDNA wiper) (Vazyme). Quantitative PCR was carried out with ChamQ Universal SYBR qPCR Master Mix (Vazyme) on an ABI StepOnePlus Real-Time PCR System (Thermo Fisher). The cycling conditions were as follows: 95°C for 30 s, followed by 40 cycles of 95°C for 10 s and 60°C for 30 s. Gene-specific primers used in this study are listed in Table S1. β-Actin was used as the internal reference gene. Relative mRNA expression levels were calculated using the 2^−ΔΔCt^ method, with normalization to β-actin and comparison to the corresponding control group. All analyses were performed in three independent experiments, each with technical triplicates, and the data were used for statistical analysis.

### Transmission electron microscopy

For ultrastructural observation, EPC cells were washed three times with PBS, detached by trypsinization, and collected into 1.5 mL microcentrifuge tubes. Cells were pelleted by centrifugation at 2,000 × *g* for 5 min and fixed in 2.5% glutaraldehyde in PBS at 4°C for 4 h. After fixation, samples were washed three times (15 min each) with PBS containing sucrose. Post-fixation was performed in 1% osmium tetroxide (OsO_4_) in PBS for 2 h at 4°C, followed by three additional washes (15 min each) with PBS–sucrose solution. Cells were then dehydrated through a graded ethanol and acetone series and infiltrated with epoxy resin. Polymerization was carried out at 37°C overnight and then at 60°C for 48 h. Ultrathin sections (70–80 nm) were cut using an ultramicrotome (UC7; Leica), collected on copper slot grids, and double-stained with 3% uranyl acetate and lead citrate for 10 min each. Sections were examined using a transmission electron microscope (HT-7800; Hitachi), and representative images were acquired for analysis of mitochondrial and nuclear ultrastructure.

### Western blotting analysis

Total cellular and membrane proteins were separated by 10% SDS-PAGE and transferred onto polyvinylidene difluoride (PVDF) membranes (Millipore). Membranes were blocked with 5% (wt/vol) skimmed milk in Tris-buffered saline containing 0.1% Tween-20 (TBST) for 1 h at room temperature and then incubated overnight at 4°C with the indicated primary antibodies. After three washes with TBST, membranes were incubated for 1 h at room temperature with horseradish peroxidase (HRP)-conjugated goat anti-rabbit or anti-mouse IgG secondary antibodies (Beyotime). Immunoreactive bands were visualized using enhanced chemiluminescence (ECL) reagents (Thermo Fisher) and imaged with a digital imaging system (Tanon). The following primary antibodies were used: anti-Bax (1:1,000, #AG1208; Beyotime), anti-α-tubulin (1:2,000, #AF2827; Beyotime), anti-Cyt-C (1:1,000, #AC908; Beyotime), anti-pro-caspase-3 (1:1,000, #AC030; Beyotime), anti-cleaved caspase-3 (1:1,000, #AC033; Beyotime), anti-pro-caspase-9 (1:1,000, #9508S; Cell Signaling Technology), anti-cleaved caspase-9 (1:1,000, #9509S; Cell Signaling Technology), anti-CHC (1:1,000, #AG1635; Beyotime), anti-TOMM20 (1:2,000, #ab186735; Abcam), and anti-Na/K-ATPase (1:2,000, #3010S; Cell Signaling Technology).

### Immunofluorescent confocal microscopy

EPC cells were seeded in 35 mm glass-bottom culture dishes and subjected to the indicated treatments. Cells were fixed with 4% paraformaldehyde in PBS for 30 min at room temperature, washed three times with PBS, and then permeabilized and blocked in PBS containing 5% goat serum, 2 mg/mL bovine serum albumin (BSA), and 0.1% Triton X-100 for 1 h at room temperature. For immunostaining, cells were incubated overnight at 4°C with primary antibodies against CHC (1:200) and/or TOMM20 (1:1,000), or with Alexa Fluor 555-conjugated Tfn (1:5,000; Invitrogen) to monitor clathrin-mediated endocytosis. After three washes with PBS containing 1% BSA, cells were incubated for 1 h at room temperature with Alexa Fluor 488- or Alexa Fluor 647-conjugated goat anti-rabbit or anti-mouse IgG secondary antibodies (1:1,000; Beyotime). Nuclei were counterstained with DAPI-containing mounting medium, and images were acquired using a confocal laser scanning microscope (Carl Zeiss, Germany).

### Statistics and reproducibility

GraphPad Prism 9.0 (GraphPad Software, San Diego, CA, USA) was used for data analysis and graph generation. Data are presented as mean ± standard deviation (SD) unless otherwise indicated. Survival curves were generated using the Kaplan–Meier method and compared by log-rank (Mantel–Cox) tests. Comparisons between two groups were performed using Student’s two-tailed *t* test, whereas comparisons involving more than two groups were analyzed by two-way analysis of variance (ANOVA) followed by appropriate *post hoc* tests. The corresponding *P* value with less than 0.05 was considered statistically significant.

## Data Availability

All data generated or analyzed during this study are included in this article and its supplemental material. No external data sets or custom code were generated or used in this study. Additional information is available from the corresponding authors upon reasonable request.

## SUPPLEMENTAL MATERIAL

Supplemental Figures and Table mbio.00453-26-s0001.docx Fig. S1 to S5 and Table S1.

Movie S1 mbio.00453-26-s0002.avi Dynamic internalization of DMSO-treated SVCV particles in EPC cells.

Movie S2 mbio.00453-26-s0003.avi CPT-treated SVCV particles remain trapped at the plasma membrane.

Movie S3 mbio.00453-26-s0004.avi Efficient entry and intracellular trafficking of SVCV in control EPC cells.

Movie S4 mbio.00453-26-s0005.avi CPT pretreatment of EPC cells immobilizes SVCV at the cell surface.

ASM does not own the copyrights to Supplemental Material that may be linked to, or accessed through, an article. The authors have granted ASM a non-exclusive, world-wide license to publish the Supplemental Material files. Please contact the corresponding author directly for reuse.

## References

[1] Naylor RL, Hardy RW, Buschmann AH, Bush SR, Cao L, Klinger DH, Little DC, Lubchenco J, Shumway SE, Troell M. 2021. A 20-year retrospective review of global aquaculture. Nature 591:551–563. doi:10.1038/s41586-021-03308-6 33762770

[2] Sanon V-P, Ouedraogo R, Toé P, El Bilali H, Lautsch E, Vogel S, Melcher AH. 2021. Socio-economic perspectives of transition in inland fisheries and fish farming in a least developed country. Sustainability 13:2985. doi:10.3390/su13052985

[3] Colombo SM, Roy K, Mraz J, Wan AHL, Davies SJ, Tibbetts SM, Øverland M, Francis DS, Rocker MM, Gasco L, Spencer E, Metian M, Trushenski JT, Turchini GM. 2023. Towards achieving circularity and sustainability in feeds for farmed blue foods. Rev Aquacult 15:1115–1141. doi:10.1111/raq.12766

[4] Mondal H, Chandrasekaran N, Mukherjee A, Thomas J. 2022. Viral infections in cultured fish and shrimps: current status and treatment methods. Aquacult Int 30:227–262. doi:10.1007/s10499-021-00795-2

[5] Ashraf U, Lu Y, Lin L, Yuan J, Wang M, Liu X. 2016. Spring viraemia of carp virus: recent advances. J Gen Virol 97:1037–1051. doi:10.1099/jgv.0.000436 26905065

[6] Ahne W, Bjorklund HV, Essbauer S, Fijan N, Kurath G, Winton JR. 2002. Spring viremia of carp (SVC). Dis Aquat Org 52:261–272. doi:10.3354/dao052261 12553453

[7] Pereiro P, Figueras A, Novoa B. 2021. Compilation of antiviral treatments and strategies to fight fish viruses. Rev Aquacult 13:1223–1254. doi:10.1111/raq.12521

[8] Owen L, Laird K, Shivkumar M. 2022. Antiviral plant-derived natural products to combat RNA viruses: targets throughout the viral life cycle. Lett Appl Microbiol 75:476–499. doi:10.1111/lam.13637 34953146 PMC9544774

[9] Yang F, Pang B, Lai KK, Cheung NN, Dai J, Zhang W, Zhang J, Chan K-H, Chen H, Sze K-H, Zhang H, Hao Q, Yang D, Yuen K-Y, Kao RY. 2021. Discovery of a novel specific inhibitor targeting influenza a virus nucleoprotein with pleiotropic inhibitory effects on various steps of the viral life cycle. J Virol 95:e01432-20. doi:10.1128/JVI.01432-20 33627391 PMC8104107

[10] Guo Q, Ban F-X, Xia W-Q, Shu Y-N, Liu Y-Q, Liu S-S, Pan L-L, Wang X-W. 2023. The essential role of clathrin-mediated endocytosis and early endosomes in the trafficking of begomoviruses through the primary salivary glands of their whitefly vectors. J Virol 97:e01067-23. doi:10.1128/jvi.01067-23 37855618 PMC10688308

[11] Battles MB, McLellan JS. 2019. Respiratory syncytial virus entry and how to block it. Nat Rev Microbiol 17:233–245. doi:10.1038/s41579-019-0149-x 30723301 PMC7096974

[12] Passioura T, Watashi K, Fukano K, Shimura S, Saso W, Morishita R, Ogasawara Y, Tanaka Y, Mizokami M, Sureau C, Suga H, Wakita T. 2018. De novo macrocyclic peptide inhibitors of hepatitis B virus cellular entry. Cell Chem Biol 25:906–915. doi:10.1016/j.chembiol.2018.04.011 29779957

[13] Schmidt AG, Lee K, Yang PL, Harrison SC. 2012. Small-molecule inhibitors of dengue-virus entry. PLoS Pathog 8:e1002627. doi:10.1371/journal.ppat.1002627 22496653 PMC3320583

[14] Teissier E, Penin F, Pécheur EI. 2010. Targeting cell entry of enveloped viruses as an antiviral strategy. Molecules 16:221–250. doi:10.3390/molecules16010221 21193846 PMC6259279

[15] Qian XJ, Zhu YZ, Zhao P, Qi ZT. 2016. Entry inhibitors: new advances in HCV treatment. Emerg Microbes Infect 5:1–8. doi:10.1038/emi.2016.3 PMC4735057 26733381

[16] Lu S, Pan X, Chen D, Xie X, Wu Y, Shang W, Jiang X, Sun Y, Fan S, He J. 2021. Broad-spectrum antivirals of protoporphyrins inhibit the entry of highly pathogenic emerging viruses. Bioorg Chem 107:104619. doi:10.1016/j.bioorg.2020.104619 33450541 PMC7784547

[17] Lu J-F, Luo S, Tang H, Liang J-H, Zhao Y-F, Hu Y, Yang G-J, Chen J. 2023. Micropterus salmoides rhabdovirus enters cells via clathrin-mediated endocytosis pathway in a pH-, dynamin-, microtubule-, rab5-, and rab7-dependent manner. J Virol 97:e00714-23. doi:10.1128/jvi.00714-23 37735152 PMC10617426

[18] Liu HB, Liu Y, Liu SL, Pang DW, Xiao GF. 2011. Clathrin-mediated endocytosis in living host cells visualized through quantum dot labeling of infectious hematopoietic necrosis virus. J Virol 85:6252–6262. doi:10.1128/JVI.00109-11 21525360 PMC3126507

[19] Shao L, Zhao J, Zhang H. 2016. Spring viraemia of carp virus enters grass carp ovary cells via clathrin-mediated endocytosis and macropinocytosis. J Gen Virol 97:2824–2836. doi:10.1099/jgv.0.000595 27590028

[20] Balmer BF, Powers RL, Zhang T-H, Lee J, Vigant F, Lee B, Jung ME, Purcell MK, Snekvik K, Aguilar HC. 2017. Inhibition of an aquatic rhabdovirus demonstrates promise of a broad-spectrum antiviral for use in aquaculture. J Virol 91:e02181-16. doi:10.1128/JVI.02181-16 27903801 PMC5286899

[21] Qiu TX, Liu L, Zhang X, Hu Y, Chen J. 2025. Antiviral activity of isoliquiritigenin against SVCV in aquaculture: a dual approach of immune modulation and viral inhibition. Aquaculture 596:741863. doi:10.1016/j.aquaculture.2024.741863

[22] Zhou Y, Qiu TX, Hu Y, Liu L, Chen J. 2022. Antiviral effects of natural small molecules on aquatic rhabdovirus by interfering with early viral replication. Zool Res 43:966–976. doi:10.24272/j.issn.2095-8137.2022.234 36257828 PMC9700502

[23] Yang F, Yang B, Song K, Jin Y, Wang G, Li P, Yu Q, Ling F. 2024. Natural product honokiol exhibits antiviral effects against Micropterus salmoides rhabdovirus (MSRV) both in vitro and in vivo. J Fish Dis 47:e13915. doi:10.1111/jfd.13915 38191774

[24] Zhang X, Guo C. 2022. Recent advances in inhibition of porcine reproductive and respiratory syndrome virus through targeting CD163. Front Microbiol 13:1006464. doi:10.3389/fmicb.2022.1006464 36187992 PMC9522899

[25] Muroi M, Lee DS. 2024. Inhibitory effects of cryptotanshinone and dihydrotanshinone i on intracellular trafficking of viral glycoproteins. J Microbiol Biotechnol 34:2457–2464. doi:10.4014/jmb.2409.09050 39726295 PMC11729303

[26] Huang C, Zhu J, Wang L, Chu A, Yin Y, Vali K, Garmendia A, Tang Y. 2020. Cryptotanshinone protects porcine alveolar macrophages from infection with porcine reproductive and respiratory syndrome virus. Antivir Res 183:104937. doi:10.1016/j.antiviral.2020.104937 32961199

[27] Mayle KM, Le AM, Kamei DT. 2012. The intracellular trafficking pathway of transferrin. BBA-GEN SUBJECTS 1820:264–281. doi:10.1016/j.bbagen.2011.09.009 PMC3288267 21968002

[28] Elkin SR, Oswald NW, Reed DK, Mettlen M, MacMillan JB, Schmid SL. 2016. Ikarugamycin: a natural product inhibitor of clathrin‐mediated endocytosis. Traffic 17:1139–1149. doi:10.1111/tra.12425 27392092 PMC5260662

[29] Sun SS, Ma SW, Li J, Zhang Q, Zhou GZ. 2023. Review on the antiviral organic agents against fish rhabdoviruses. Fishes 8:57. doi:10.3390/fishes8010057

[30] Liao W, Huang L, Han S, Hu D, Xu Y, Liu M, Yu Q, Huang S, Wei D, Li P. 2022. Review of medicinal plants and active pharmaceutical ingredients against aquatic pathogenic viruses. Viruses 14:1281. doi:10.3390/v14061281 35746752 PMC9230652

[31] Zhou Y, Qiu TX, Hu Y, Ji J, Liu L, Chen J. 2022. Evaluation on the antiviral activity of artemisinin against rhabdovirus infection in common carp. Aquaculture 559:738410. doi:10.1016/j.aquaculture.2022.738410

[32] Shen YF, Liu YH, Li BY, Liu TQ, Wang GX. 2020. Evaluation on antiviral activity of a novel arctigenin derivative against multiple rhabdoviruses in aquaculture. Virus Res 285:198019. doi:10.1016/j.virusres.2020.198019 32417180

[33] Qiu T-X, Wang H, Hu Y, Shan L-P, Liu G-L, Liu L, Chen J. 2025. Inhibition of fish rhabdovirus demonstrates application prospect of two methylimidazole phenylpropanoid-based small molecules in aquaculture. Aquaculture 595:741636. doi:10.1016/j.aquaculture.2024.741636

[34] Silva-Júnior EF da. 2022. Entry Inhibitors of RNA Viruses. Curr Med Chem 29:609–611. doi:10.2174/092986732904220207113503 35227179

[35] Pattnaik GP, Chakraborty H. 2020. Entry inhibitors: efficient means to block viral infection. J Membr Biol 253:425–444. doi:10.1007/s00232-020-00136-z 32862236 PMC7456447

[36] Otręba M, Kośmider L, Rzepecka-Stojko A. 2020. Antiviral activity of chlorpromazine, fluphenazine, perphenazine, prochlorperazine, and thioridazine towards RNA-viruses. a review. Eur J Pharmacol 887:173553. doi:10.1016/j.ejphar.2020.173553 32949606 PMC7493736

[37] Piccinotti S, Whelan SPJ. 2016. Rabies internalizes into primary peripheral neurons via clathrin coated pits and requires fusion at the cell body. PLoS Pathog 12:e1005753. doi:10.1371/journal.ppat.1005753 27463226 PMC4963122

[38] Wang S, Huang X, Huang Y, Hao X, Xu H, Cai M, Wang H, Qin Q. 2014. Entry of a novel marine DNA virus, Singapore grouper iridovirus, into host cells occurs via clathrin-mediated endocytosis and macropinocytosis in a pH-dependent manner. J Virol 88:13047–13063. doi:10.1128/JVI.01744-14 25165116 PMC4249105

[39] Huang R, Zhu G, Zhang J, Lai Y, Xu Y, He J, Xie J. 2017. Betanodavirus-like particles enter host cells via clathrin-mediated endocytosis in a cholesterol-, pH- and cytoskeleton-dependent manner. Vet Res 48:8. doi:10.1186/s13567-017-0412-y 28179028 PMC5299686

[40] Bayati A, Kumar R, Francis V, McPherson PS. 2021. SARS-CoV-2 infects cells after viral entry via clathrin-mediated endocytosis. J Biol Chem 296:100306. doi:10.1016/j.jbc.2021.100306 33476648 PMC7816624

[41] Vigant F, Santos NC, Lee B. 2015. Broad-spectrum antivirals against viral fusion. Nat Rev Microbiol 13:426–437. doi:10.1038/nrmicro3475 26075364 PMC4554337

[42] Bello-Onaghise G, Wang G, Han X, Nsabimana E, Cui W, Yu F, Zhang Y, Wang L, Li Z, Cai X, Li Y. 2020. Antiviral strategies of chinese herbal medicine against PRRSV infection. Front Microbiol 11:1756. doi:10.3389/fmicb.2020.01756 32849384 PMC7401453

[43] Chen X, Feng H, Wang L, Zhang J, Lu X, Xu G, Liu S, Yang Q, Feng X, Wang J, Zhang K, Li J. 2025. Cryptotanshinone suppresses BVDV propagation by suppressing cell apoptosis and restoring hormone secretion in bovine granulosa cells. Viruses 17:1433. doi:10.3390/v17111433 41305456 PMC12656771

[44] Ju PM, Ma SW, Li YY, Zhang SF, Li J, Zhou GZ. 2024. Investigation of the antiviral mechanism of curcumin analog EF-24 against Siniperca cachuatsi rhabdovirus. Fishes 9:179. doi:10.3390/fishes9050179

[45] Ortega-Villaizan M del M, Chico V, Perez L. 2022. Fish innate immune response to viral infection—an overview of five major antiviral genes. Viruses 14:1546. doi:10.3390/v14071546 35891526 PMC9317989

[46] Kobryń J, Dałek J, Musiał W. 2021. The influence of selected factors on the aqueous cryptotanshinone solubility. Pharmaceutics 13:992. doi:10.3390/pharmaceutics13070992 34209049 PMC8309180

[47] Zhao W, Ruan B, Sun X, Yu Z. 2023. Preparation and optimization of surface stabilized cryptotanshinone nanocrystals with enhanced bioavailability. Front Pharmacol 14:1122071. doi:10.3389/fphar.2023.1122071 36817118 PMC9935824

[48] Zhang X-X, Cao Y-F, Wang L-X, Yuan X-L, Fang Z-Z. 2017. Inhibitory effects of tanshinones towards the catalytic activity of UDP-glucuronosyltransferases (UGTs). Pharm Biol 55:1703–1709. doi:10.3109/13880209.2015.1045621 28466663 PMC6130658

[49] Chen ZY, Liu H, Li ZQ, Zhang QY. 2008. Development and characterization of monoclonal antibodies to spring viraemia of carp virus. Vet Immunol Immunopathol 123:266–276. doi:10.1016/j.vetimm.2008.02.011 18378003

[50] Liu L, Qiu TX, Song DW, Shan LP, Chen J. 2020. Inhibition of a novel coumarin on an aquatic rhabdovirus by targeting the early stage of viral infection demonstrates potential application in aquaculture. Antivir Res 174:104672. doi:10.1016/j.antiviral.2019.104672 31825851

