# Supplementary material for: A novel host-targeted inhibitor of clathrin-mediated endocytosis limits spring viremia of carp virus infection
Source: mBio. 2026 May 29;17(7):e00453-26. doi: 10.1128/mbio.00453-26 (PMC13344028; doi:10.1128/mbio.00453-26)
Supplement: Supplemental Figures and Table — Fig. S1 to S5 and Table S1. [file mbio.00453-26-s0001.docx]

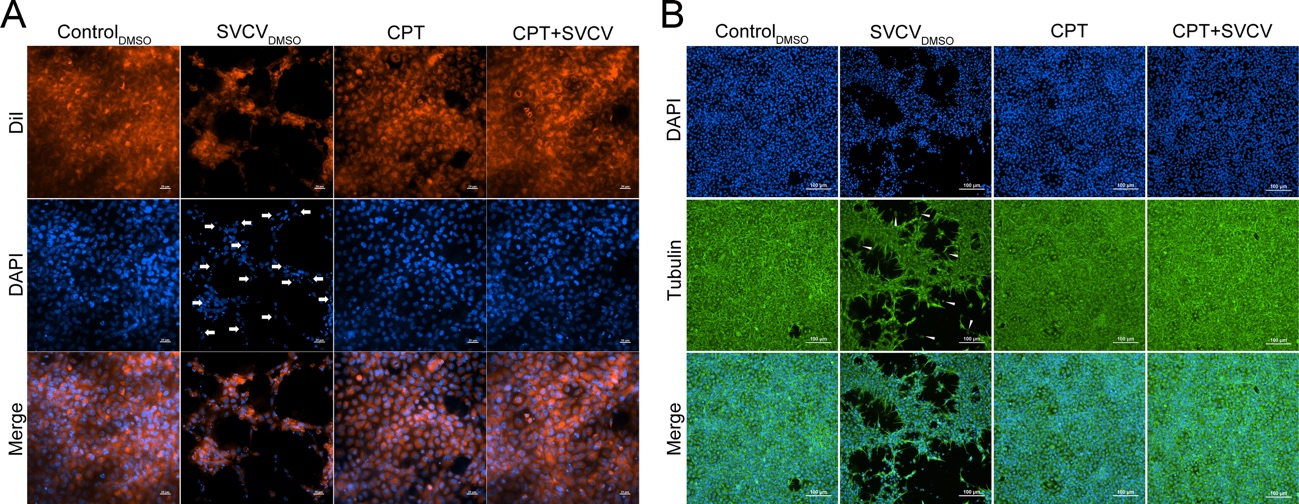


**FIG S1. CPT alleviates SVCV-induced nuclear damage and cytoskeletal collapse in EPC cells.** (A) DAPI and DiI staining of EPC cells treated with DMSO (Control_DMSO_), SVCV_DMSO_, CPT, or CPT+SVCV for 48 h. Arrows indicate condensed and fragmented nuclei characteristic of apoptosis in SVCV-infected cells, which are largely absent in CPT-treated infected cells. Scale bars, 20 μm. (B) Immunofluorescence staining of nuclei (DAPI) and microtubules (tubulin) under the same conditions. SVCV infection causes disruption and aggregation of the microtubule network (arrowheads), whereas CPT preserves a dense, radial cytoskeletal organization similar to that in control cells. Scale bars, 100 μm.

**
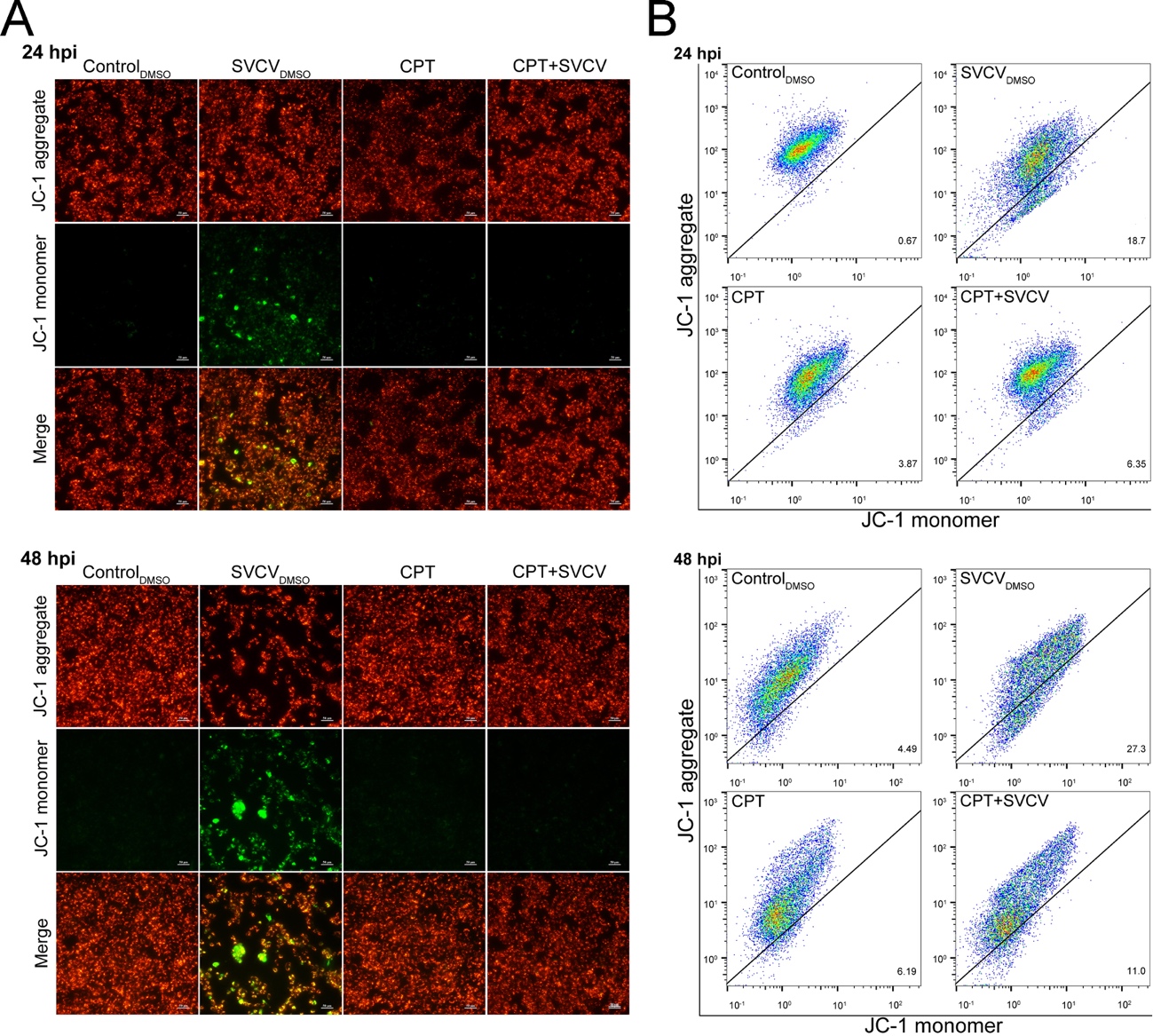
**

**FIG S2. CPT attenuates SVCV-induced loss of mitochondrial membrane potential in EPC cells.** (A) JC-1 staining of EPC cells treated with DMSO, SVCV_DMSO_, CPT, or CPT+SVCV at 24 and 48 hpi. Red fluorescence indicates JC-1 aggregates and green fluorescence indicates JC-1 monomers. SVCV infection increases green fluorescence, whereas CPT largely preserves red fluorescence and reduces JC-1 monomer signal. Scale bars, 20 μm. (B) Flow-cytometric analysis of JC-1 fluorescence at 24 and 48 hpi. SVCV shifts the cell population toward higher JC-1 monomer and lower aggregate signal, consistent with mitochondrial depolarization, while CPT reverses this shift in infected cells.

**
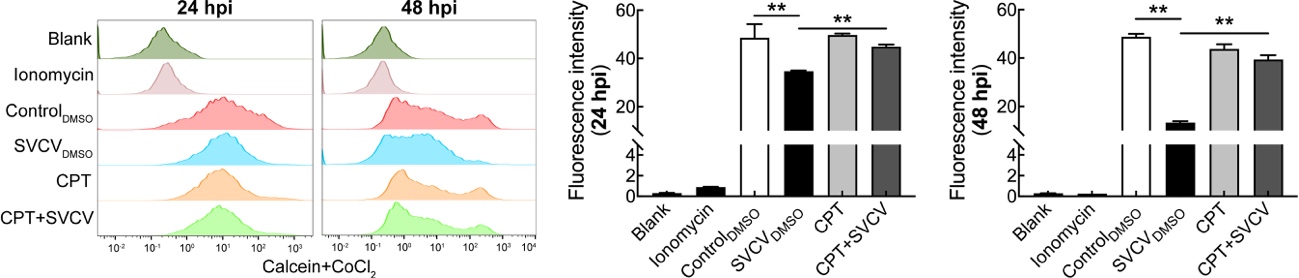
**

**FIG S3. CPT restores mitochondrial calcein fluorescence reduced by SVCV infection.** Flow-cytometric histograms (left) showing calcein/CoCl_2_ fluorescence in EPC cells treated with Blank, ionomycin, Control_DMSO_, SVCV_DMSO_, CPT, or CPT+SVCV at 24 and 48 hpi. Ionomycin and SVCV markedly decrease calcein fluorescence, whereas CPT alone has little effect and significantly restores fluorescence in SVCV-infected cells. Bar graphs (right) summarize mean fluorescence intensity at 24 and 48 hpi. Data are presented as mean ± SD. Statistical significance was determined by one-way ANOVA with appropriate post hoc tests; ***p* < 0.01.

**
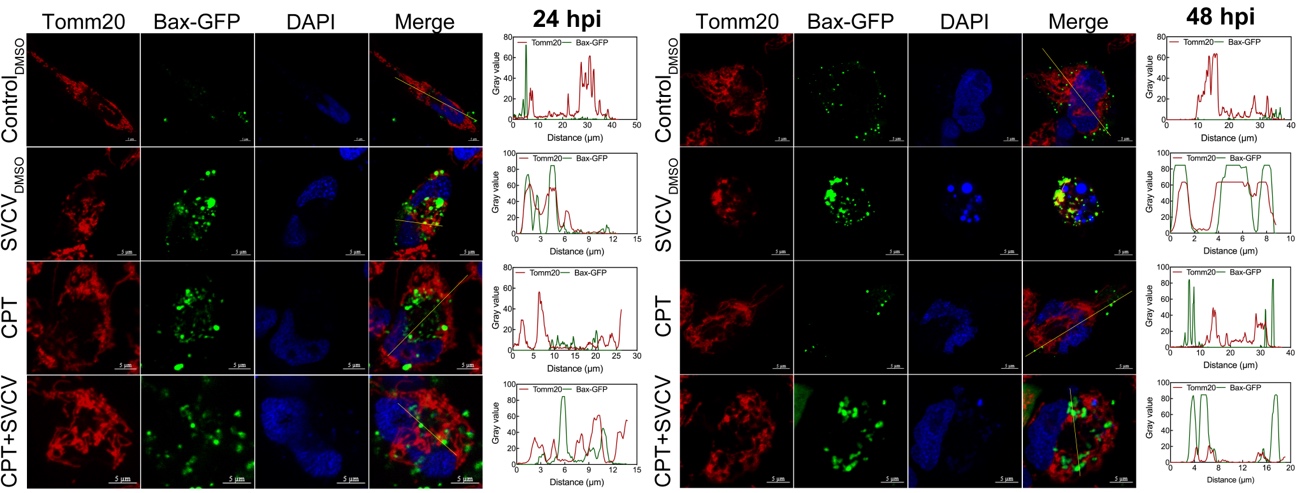
**

**FIG S4. CPT inhibits SVCV-induced Bax recruitment to mitochondria.** Representative confocal images of EPC cells expressing Bax-GFP (green) and stained for the mitochondrial marker Tomm20 (red) and nuclei (DAPI, blue) at 24 hpi (left panel set) and 48 hpi (right panel set). In SVCV_DMSO_ cells, Bax-GFP strongly colocalizes with Tomm20, indicating Bax translocation to mitochondria, whereas CPT treatment greatly reduces this colocalization. Line-scan plots (right of each image set) show fluorescence intensity profiles of Tomm20 and Bax-GFP along the indicated lines. Scale bars, 5 μm.


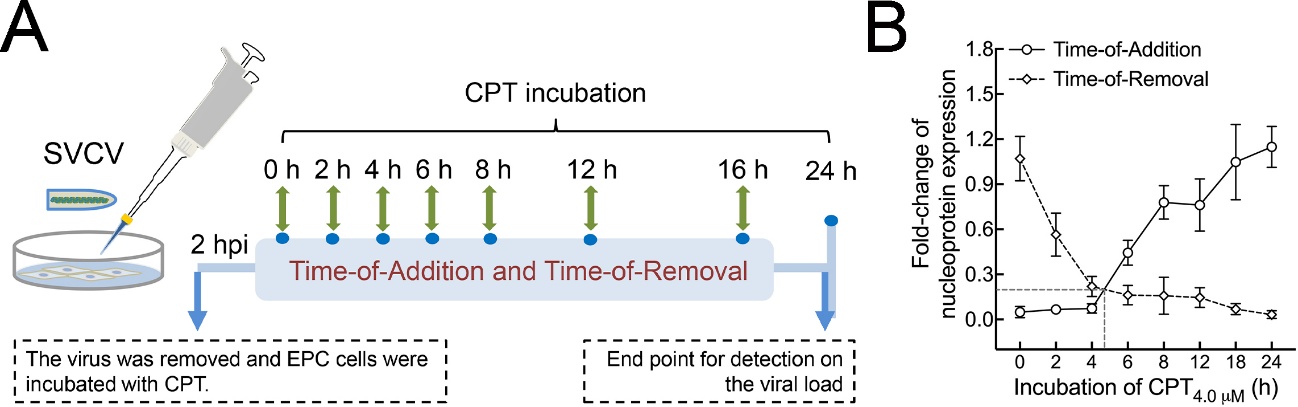


**FIG S5. Time-of-addition and time-of-removal analysis of CPT during SVCV infection.** (A) Schematic of the experimental design. EPC cells were infected with SVCV, the inoculum was removed at 2 hpi, and CPT (4 μM) was either added or removed at the indicated time points up to 24 h. Viral load was measured at the endpoint. (B) Time-of-addition and time-of-removal curves showing the effect of CPT on SVCV nucleoprotein expression. The intersection of the two curves between 4–6 h indicates that CPT primarily targets early stages of infection. Data are presented as mean ± SD.

**Table S1** Sequences of primer pairs used for the analysis of gene expression by qPCR.

| Genes | | Primer Sequence from 5’-3’ |
| --- | --- | --- |
| SVCV *nucleoprotein* (SVCV-*N*) | Forward | AACAGCGCGTCTTACATGC |
|  | Reserve | CTAAGGCGTAAGCCATCAGC |
| *ifn1* | Forward | ACCAAACCCAAATGTGGACGTG |
|  | Reserve | CCACTCATTTCCCGAAGCAGA |
| *ifn2* | Forward | GATGAAGGTGCCATTTCCAAG |
|  | Reserve | CACTGTCGTTAGGTTCCATTGCTC |
| *isg15* | Forward | AAGCCATATTCAGCGAAGC |
|  | Reserve | AACCGTTATCGGCAGACAG |
| *mx1* | Forward | ATGAATCCTGGAAGCCCTC |
|  | Reserve | GAACTTCGGGAAGAATTTGC |
| *β-actin* (EPC cells) | Forward | GCTATGTGGCTCTTGACTTCGA |
|  | Reserve | CCGTCAGGCAGCTCATAGCT |
| *β-actin* (common carp) | Forward | GATGATGAAATTGCCGCACTG |
|  | Reserve | ACCAACCATGACACCCTGATGT |
